# The CEA^−/lo^ colorectal cancer cell population harbors cancer stem cells and metastatic cells

**DOI:** 10.18632/oncotarget.13029

**Published:** 2016-11-02

**Authors:** Chang Yan, Yibing Hu, Bo Zhang, Lei Mu, Kaiyu Huang, Hui Zhao, Chensen Ma, Xiaolan Li, Deding Tao, Jianping Gong, Jichao Qin

**Affiliations:** ^1^ Department of Surgery, Tongji Hospital, Tongji Medical College, Huazhong University of Science and Technology, Wuhan, China; ^2^ Molecular Medicine Center, Tongji Hospital, Tongji Medical College, Huazhong University of Science and Technology, Wuhan, China

**Keywords:** colorectal cancer, carcinoembryonic antigen, cancer stem cell, metastasis

## Abstract

Serum carcinoembryonic antigen (CEA) is the most commonly used tumor marker in a variety of cancers including colorectal cancer (CRC) for tumor diagnosis and monitoring. Recent studies have shown that colonic crypt cells expressing little or no CEA may enrich for stem cells. Numerous studies have clearly shown that there exist CRC patients with normal serum CEA levels during tumor progression or even tumor relapse, although CEA itself is considered to promote metastasis and block cell differentiation. These seemingly contradictory observations prompted us to investigate, herein, the biological properties as well as tumorigenic and metastatic capacity of CRC cells that express high (CEA^+^) versus low CEA (CEA^−/lo^) levels of CEA. Our findings show that the abundance of CEA^−/lo^ cells correlate with poor differentiation and poor prognosis, and moreover, CEA^−/lo^ cells form more spheres *in vitro*, generate more tumors and exhibit a higher potential in developing liver and lung metastases than corresponding CEA^+^ cells. Applying RNAi-mediated approach, we found that IGF1R mediated tumorigenic and capacity of CEA^−/lo^ cells but did not mediate those of CEA^+^ cells. Notably, our data demonstrated that CEA molecule was capable of protecting CEA^−/lo^ cells from anoikis, implying that CEA^+^ cells, although themselves possessing less tumorigenic and metastatic capacity, may promote metastasis of CEA^−/lo^ cells via secreting CEA molecule. Our observations suggest that, besides targeting CEA molecule, CEA^−/lo^ cells may represent a critical source of tumor progression and metastasis, and should therefore be the target of future therapies.

## INTRODUCTION

Colorectal cancer (CRC) is the third most common cause of death from cancer [1]. CRC is heterogeneous, manifesting variegated cellular morphologies and histopathological presentations. New insights into tumor heterogeneity may help to devise novel diagnostic and therapeutic procedures.

Serum carcinoembryonic antigen (CEA) is recommended as a tumor marker in colorectal cancer (CRC) for tumor detecting and monitoring response to therapy [2]. It is characterized as a member of CD66 cluster of differentiation and several studies have provided evidence that CEA protein blocks cell differentiation and thus promote tumor progression [3, 4]. However, recent studies have clearly demonstrated that colonic cells expressing little or no CEA (i.e., CEA^−/lo^ cells) locate in the lower levels of the crypts and normal stem cells that expressing Lgr5 are similarly confined to the bottom of crypts [5, 6], implying that CEA^−/lo^ cells may enrich stem cells. Furthermore, well-differentiated CRCs produce more CEA in serum and primary tissues than poorly differentiated specimens [7, 8]. Therefore, it is not defined whether free CEA protein or cellular CEA or both take effects in cell differentiation.

As a member of immunoglobulin supergene family, CEA is involved in intercellular adhesion and thus mediates homotypic cell aggregation [9]. Additional evidence has showed that forced overexpression of CEA is associated with anoikis, a form of apoptosis caused by detachment from cell matrix, and therefore enhances metastasis [10]. However, it has been challenged by the evidence that CEA mRNA expression in primary tumors is higher than in liver metastases and inversely correlates with the number of metastatic lymph nodes [11]. Actually, metastasis is a complex cascade, besides being resistant to anoikis, cells must acquire the ability to migrate, invade, and initiate tumors in foreign sites [12, 13]. These studies raised a fundamental question: could CEA^−/lo^ CRC cells intrinsically distinct from CEA^+^ cells and thus play differential roles in tumor initiating, differentiation and metastasis? Herein, we addressed these clinically relevant questions by separating bulk CRC cells into CEA^−/lo^ and CEA^+^ subpopulation.

## RESULTS

### Increased CEA^−/lo^ cells positively correlate with tumor grade and poor prognosis in CRC

**Figure 1:**
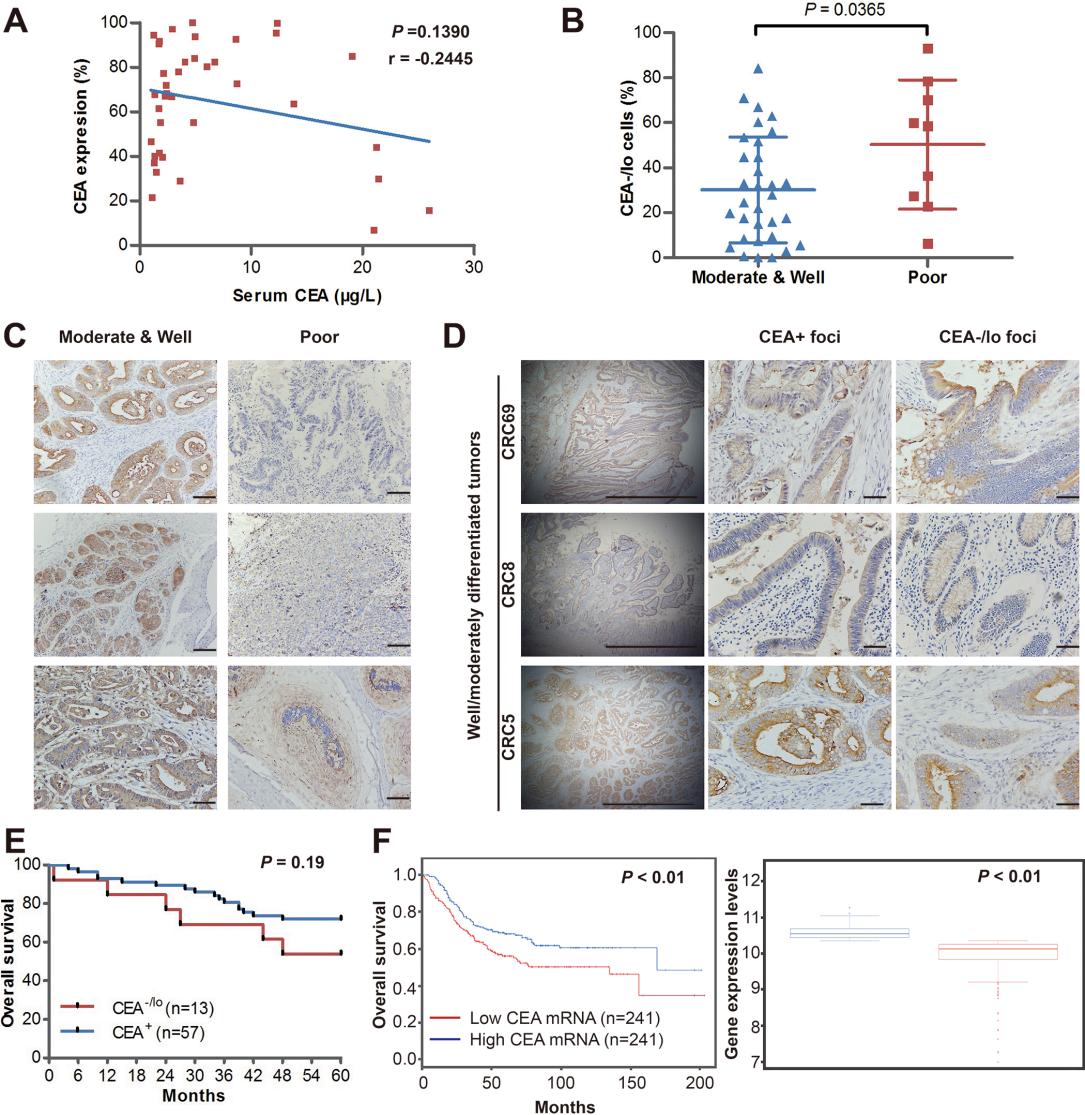
CEA^−/lo^ cells are abundant in high-grade CRC tumors and positively correlate with poor prognosis (**A**) Regression analysis of serum levels of CEA and tissue CEA expression. Plots of serum and tissue CEA for all patients (*n* = 40), regression line and *P* value were indicted. r = −0.445. (**B**) The percentage of CEA^−/lo^ CRC cells in well/moderately and poorly differentiated CRC. (**C**) Representative microphotographs of CEA staining in well, moderately and poorly differentiated CRC tumors. Scale bars: 100 μm. (**D**) Representative microphotographs of CEA staining of CEA^+^ foci and CEA^−/lo^ foci in well/moderately differentiated CRC tumors. Scale bars: 100μm. (**E**) Kaplan-Meier analysis of overall survival for 70 newly diagnosed CRC patients according to IHC scores of CEA staining. (**F**) Survival analysis of 482 diagnosed CRC patients based on CEA mRNA levels in *SurvExpress* colon metabase (left panel). The CEA mRNA levels of both risk groups were indicated (right panel).

**Figure 2:**
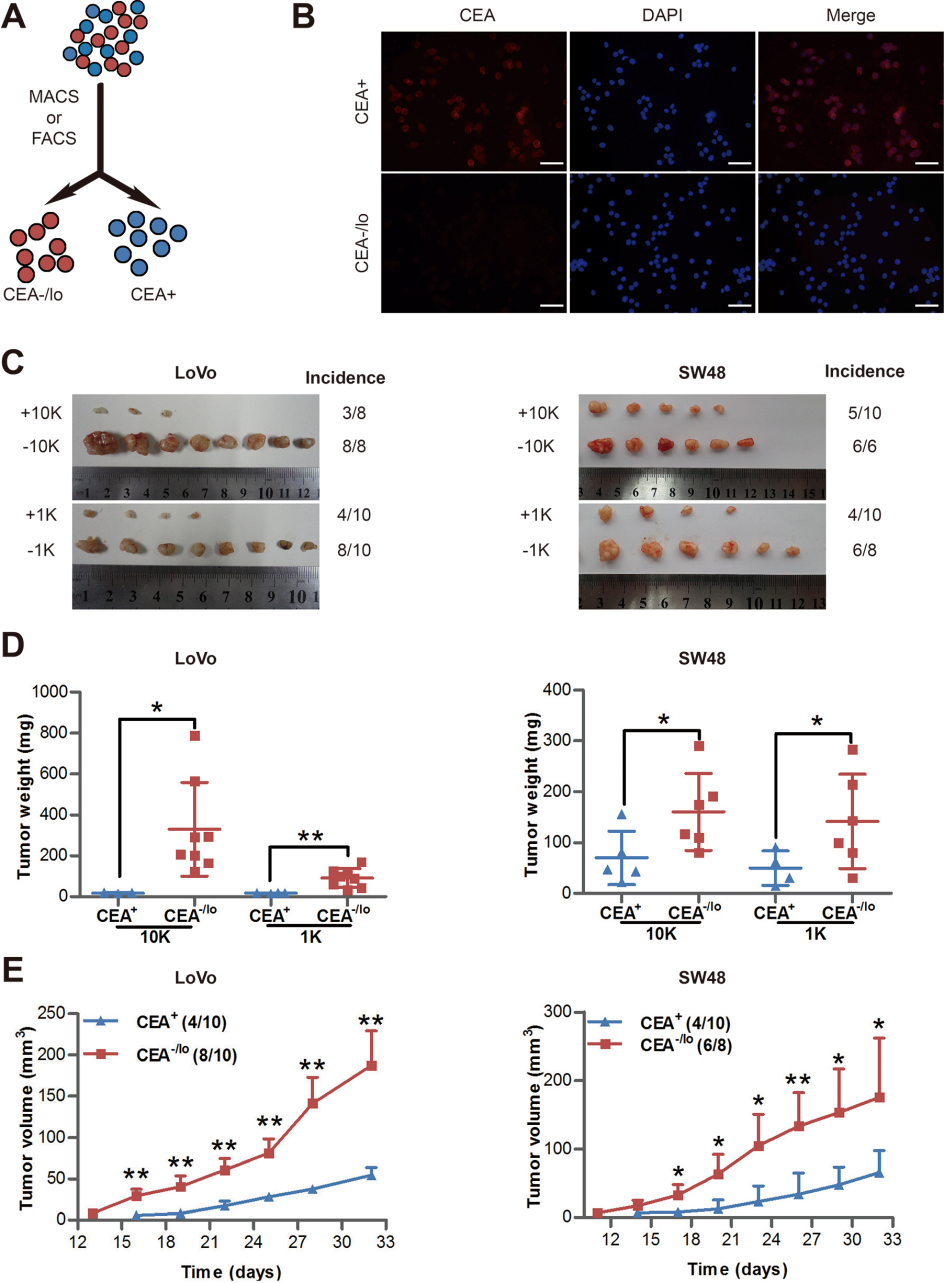
Tumorigenic capacity of CEA^+^ and CEA^/lo^ cells purified from LoVo and SW48 cells (**A**) Schematic of CEA^+^ and CEA^−/lo^ cell sorting. (**B**) Immunofluorescence staining of CEA in purified CEA^+^ and CEA^−/lo^ LoVo cells. Scale bars: 100 μm. (**C**–**E**) Limiting dilution assays estimating tumor incidence, tumor weights and tumor volumes of CEA^+^/CEA^−/lo^ LoVo and SW48 cells. Tumors were harvested at 32 days postimplantation and represented images were taken (C), tumor weights were measured (D) and tumor volumes were measured in mice with 1,000 cells injection (E). Data are presented as mean ± SD; *P < 0.05, **P < 0.01.

Serum CEA has been recommended as a diagnostic and prognostic indicator of colorectal cancer [2]. We first studied the correlations of preoperative serum CEA, quantified proportions of CEA^+^/CEA^−/lo^ CRC cells and tumor grade. Regression analysis yielded no evidence of correlation of serum CEA and tissues CEA expression (*n* = 40), indicating that elevated serum CEA do not necessarily connote elevated tumor tissue levels of CEA (*r* = −0.2445 and *P* > 0.05, Figure 1A and Supplementary Table S1). This may explain controversial roles of serum CEA and tumor tissue CEA in tumor characterization and prognosis. Quantification revealed significantly increased proportions of CEA^−/lo^ cells in poorly differentiated CRC tumors compared to well/moderately differentiated CRC tumors (Figure 2B and Supplementary Table S1). We also performed a semi-quantitative CEA immunohistochemical analysis on CRC tumor tissue (*n* = 70). Consistent with FACS results, 40.0% (6/15) patients with poorly differentiated tumors had low CEA expression in tumor specimens while 12.7% (7/55) patients had low CEA expression in well/moderately differentiated tumors (Table 1). In well/moderately differentiated tumors, the main histological pattern was differentiated areas with glandular structures represented the primary histological pattern and most CRC cells were stained strongly positive for CEA, however, many tumor cells were lacking CEA expression in poorly differentiated tumors (Figure 1C). More interesting, intra-tumor heterogeneity of CEA expression also contributes to tumor-cell differentiation. In well/moderately differentiated tumors, the CEA content was high on the tumor cell surface within lower tumor grade glands while tumor cells were absent or low with CEA expression in poorly-differentiated areas (Figure 1D). These data further support increased proportions CEA^−/lo^ cells are positively correlated with degree of tumor grade.

**Table 1:** Correlations between CEA expression and clinicopathological factors in CRCs

	CEA score		P value
	> 4	≤ 4	
Gender			
Male	36	7	0.54
Female	21	6	
Age Median (SD)	57.68 (13.42)	57.54 (17.21)	0.97
Tumor grade			
I & II	48	7	0.03
III	9	6	
Duke's stage			
A	13	1	0.50
B	25	8	
C	17	4	
D	2	0	

NOTE. Immunohistochemical analysis were performed on formalin-fixed paraffin-embedded human CRC sections. CEA levels were evaluated according to immunoreactive scores (IRS). Patients were categorized into two group based on IRS. *P* valves are listed comparing two categories at different clinicopathological factors using Fisher's exact test.

Finally, we assessed the association of CEA expression in CRC tumor tissue with clinical outcome. Our immunohistochemical analysis of above CRC samples (*n* = 70) showed patients with lower CEA expression had more reduced survival than patients with higher CEA expression, though, maybe due to limited patient tumor sample number, there was no significant difference (Figure 1E). To further confirm the prognostic performance of CEA expression, we employed *SurvExpress*, an online biomarker validation tool and database, for survival analysis [14]. Analysis of multiple microarray datasets revealed that reduced tumor CEA mRNA levels were positively correlated with shortened patient survival (Figure 1F and Supplementary Figure S1). Together, these data suggest that CEA^−/lo^ cells positively correlate with tumor grade and poor prognosis in CRC.

### CEA^−/lo^ LoVo and SW48 cells possess high tumorigenic capacity

To explore whether cells lacking CEA expression is intrinsic different from CEA^+^ cells in tumorigenic capacity, we carried out immunostaining for CEA in CRC cell lines (i.e., LoVo and SW48 cells) with anti-CEA antibody, and then employed MACS and/or FACS to acutely purify out CEA^+^ and CEA^−/lo^ CRC cells (Figure 2A). Immunofluorescence staining or post-sorting analysis of purified cells confirmed that most purified CEA^+^ cells stained strongly positive for CEA protein, whereas CEA^−/lo^ cells were week or negative for CEA (Figure 2B and Supplementary Figure S2). Next, we examined *in vivo* tumorigenicity of both subpopulations with limiting-dilution assays (LDAs) by monitoring tumor latency, incidence, growth rate and endpoint weight. We implanted 10,000 and 1,000 each of CEA^+^ and CEA^−/lo^ LoVo and SW48 cells in female BALB/c-nu mice. Surprisingly, CEA^−/lo^ LoVo cells and SW48 cells demonstrated higher tumor initiating capacity (Figure 2C–2D, Table 2 and Supplementary Table S2) and developed larger tumors (Figure 2C–2E). Tumor latency and growth rates also showed similar pattern: CEA^−/lo^ cells initiated tumors 3 days earlier and grew faster than corresponding CEA^+^ cells (Figure 2E and Supplementary Table S2). These data revealed that CEA^−/lo^ cells derived from long-term cultured CRC cell lines possessed higher tumorigenic capacity than CEA^+^ cells.

**Table 2:** Tumor-initiating frequency of CEA^+^ and CEA^−/lo^ CRC cells in Balb/c-nu mice or NOD/SCID mice

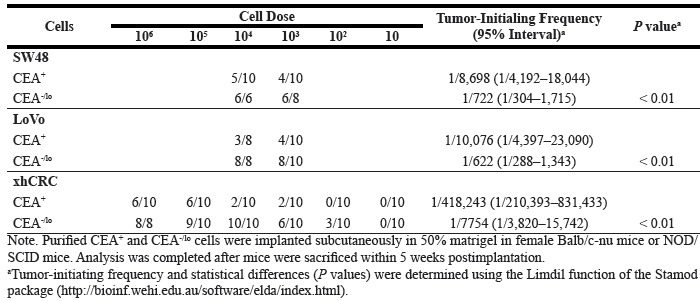

### CEA^−/lo^ cells derived from xenografts enrich tumor-initiating cells

**Figure 3:**
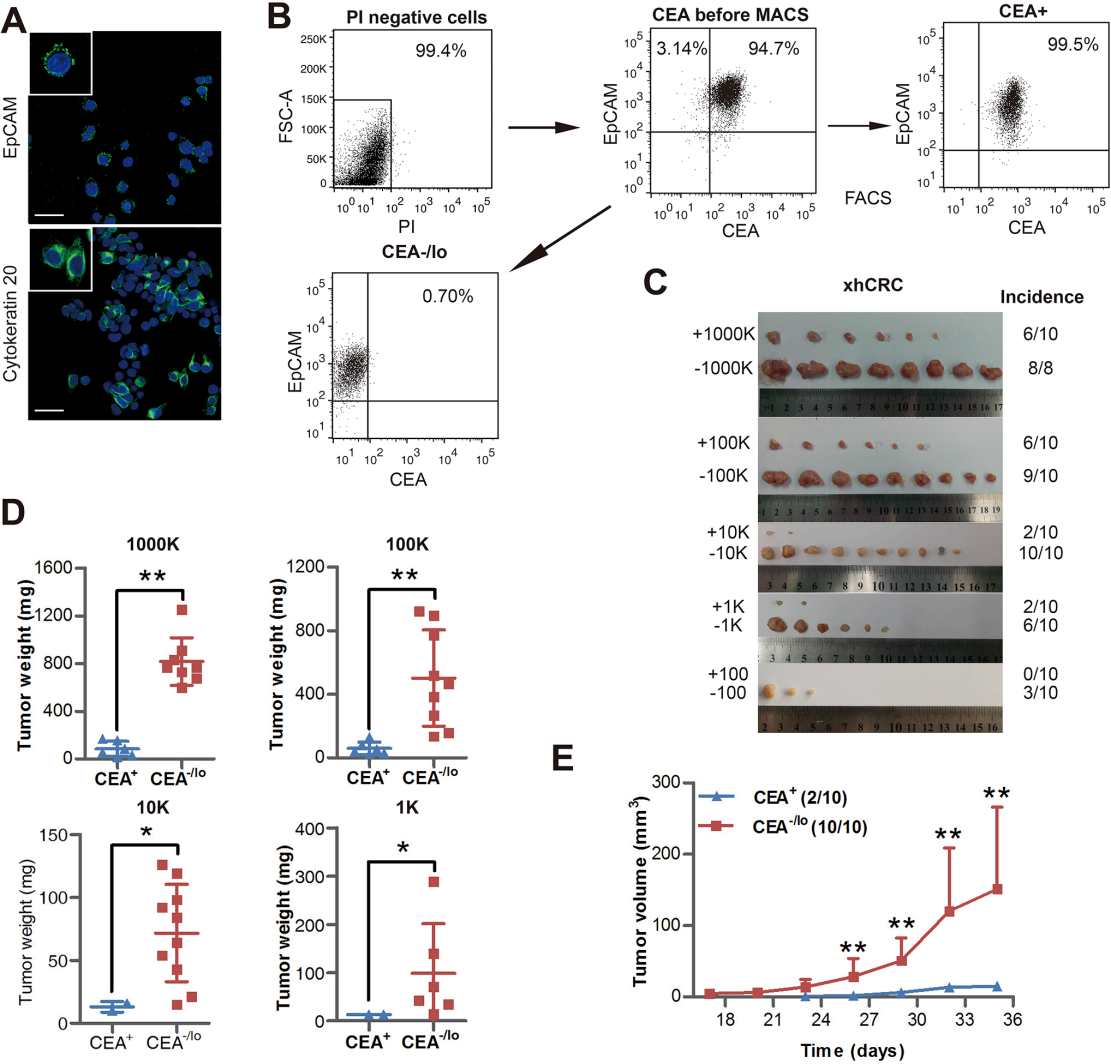
Tumorigenic capacity of CEA^+^ and CEA^/lo^ xhCRC cells (**A**) Representative confocal microscopy analysis of EpCAM and cytokratin 20 in xhCRC cells. Nuclei are stained in blue. Scale bars: 30 μm. (**B**) An example of post-sorting analysis of purified CEA^+^ and CEA^−/lo^ xhCRC cells. (**C**–**E**) CEA^+^ and CEA^−/lo^ xhCRC cells were acutely purified and then implanted subcutaneously in female NOD/SCID mice; different cell dosages (i.e., 1,000K, 100K, 10K, 1K, 100/injection) were applied. Represent images were taken and tumor incidence was indicated (C), tumor weights were measured (D) and tumor volumes were measured in mice with 10,000 cells injection starting from 17 days postimplantation (E). Data are presented as mean ± SD; *P < 0.05, ** *P* < 0.01.

Recent studies have shown that patient-derived colorectal cancer xenografts (PDXs) are good tools, which may faithfully report therapeutic response in patients and are widely used in cancer research fields, in particular, CSC studies [15]. We therefore established a xenograft tumor (xhCRC) in female NOD/SCID mice using a primary tumor derived from a female CRC patient with Dukes' C stage. Immunofluorescence staining revealed that xhCRC cells were positive for EpCAM and cytokeratin 20, indicating that xhCRC cells were epithelial cells that originated from human CRC tumors (Figure 3A). To purify CEA^+^ and CEA^−/lo^ xhCRC cells, xenograft tumors were processed into single cells, and then cells that were PI negative were sorted out based on CEA^+^EpCAM^+^ to obtain CEA^+^ xhCRC cells and CEA^−/lo^EpCAM^+^ to acquire CEA^−/lo^ xhCRC cells (Figure 3B). We then performed LDAs by implanting 1,000,000, 100,000, 10,000, 1,000, 100 and 10 of CEA^+^ and CEA^−/lo^ xhCRC cells subcutaneously into female NOD/SCID mice. Consistent with the findings in CRC cell lines, CEA^−/lo^ xhCRC cells demonstrated higher tumor-initiating capacity (Figure 3C–3D, Table 2 and Supplementary Table S2) and developed larger tumors than CEA^+^ cells (Figure 3C–3E and Supplementary Table S2). More significantly, we found that CEA^−/lo^ xhCRC cells initiated tumors 6 days earlier than CEA^+^ xhCRC cells (Figure 3E and Supplementary Table S2). While 100 CEA^+^ xhCRC cells did not initiate tumors (0/10, 0%), 100 CEA^−/lo^ xhCRC cells initiated tumors (3/10, 30%), indicating that CEA^−/lo^ xhCRC cells enrich tumor-initiating cells (Figure 3C and Table 2). Taken together, these data strongly indicate that CEA^−/lo^ xhCRC cells harbor tumor-initiating cells.

### CEA^−/lo^ CRC cells exhibit cancer stem-like features

**Figure 4:**
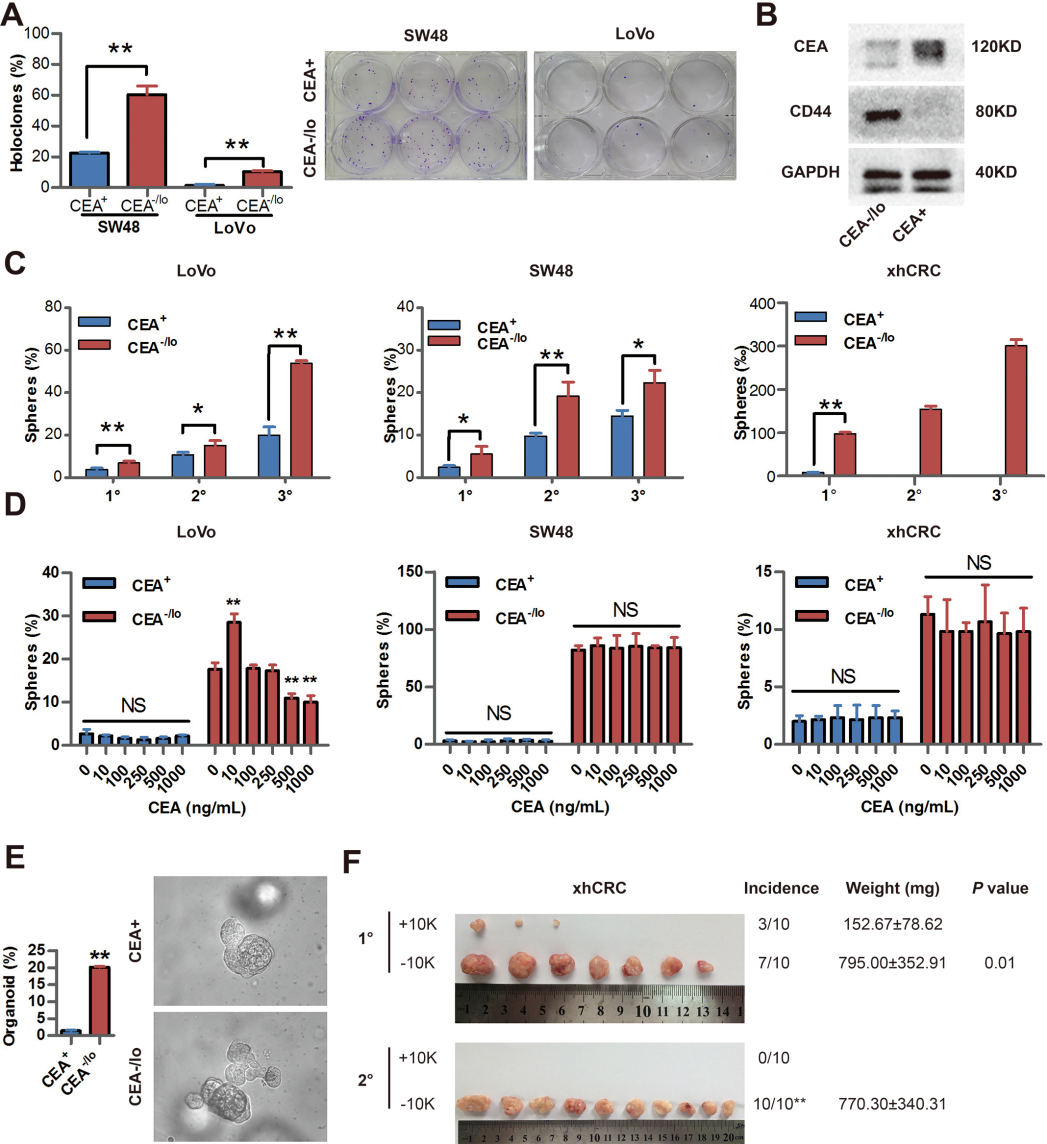
CEA^−/lo^ CRC cells exhibit cancer stem-like features (**A**) Clonal culture of CEA^+^ and CEA^−/lo^ SW48, LoVo cells. *P < 0.01 (left panel). Representative images of holoclones were taken (right panel). (**B**) Representative immunoblot analysis of CEA and CD44 in CEA^+^ and CEA^−/lo^ xhCRC cells. Loading control was assessed by GAPDH. (**C**) Serial sphere-formation assays of CEA^+^ and CEA^−/lo^ SW48, LoVo, xhCRC cells. *P < 0.05, **P < 0.01. (**D**) Sphere-formation assays of CEA^+^ and CEA^−/lo^ SW48, LoVo, xhCRC cells treated with different doses of exogenous CEA molecule. *P < 0.05, **P < 0.01, compared to corresponding cells with 0 ng/mL CEA. (**E**) Organoid culture assay of CEA^+^ and CEA^−/lo^ LoVo cells. *P < 0.05 (**F**) Serial transplantation assays of CEA^+^ and CEA^−/lo^ xhCRC cells. For each generation, tumor images, tumor incidence (**P < 0.01, Fisher exac*t* test), tumor weights (mean ± SD), and *P* values (Student *t*-test) were indicated.

In order to evaluate self-renewal capacity of CEA^+^ and CEA^−/lo^ cells, we performed several *in vitro* assays. CEA^−/lo^ Lovo and SW48 cells formed more holoclones than the corresponding CEA^+^ cells (Figure 4A), suggesting that CEA^−/lo^ cells enrich more holoclone-forming cells since holoclones are shown to enrich CSCs [16]. Western blotting revealed high protein level of CD44, a cancer stem cell marker, in CEA^−/lo^ CRC cells (Figure 4B). When cultured with stem cell medium in ultra-low attachment plates, CEA^−/lo^ LoVo and SW48 cells initiated more spheres than CEA^+^ cells in 1° generation, and more significantly, in serial sphere-formation assays, CEA^−/lo^ cell-originated spheres regenerated more and larger secondary and tertiary spheres than CEA^+^ cell-originated spheres (Figure 4C and Supplementary Figure S3). In agreement with these findings, CEA^−/lo^ xhCRC cells could be passaged for at least three generations and showed an increased sphere-propagating capacity, whereas CEA^+^ xhCRC cells only initiated much fewer spheres and could not regenerate secondary spheres (Figure 4C and Supplementary Figure S3). We then investigated self-renewing capacity of CEA^+^ and CEA^−/lo^ subsets upon soluble CEA treatments. Surprisedly, CEA enhanced self-renewal capacity of CEA^−/lo^ LoVo cells at a concentration of 10ng/mL, while inhibited CEA^−/lo^ LoVo cells at high concentrations (500ng/mL or 1000ng/mL), though free CEA molecule took no effects on CEA^+^ LoVo cells (Figure 4D). And, soluble CEA did not affect either CEA^+^ or CEA^−/lo^ cells in SW48 cells or xhCRC (Figure 4D). These results suggested whether soluble CEA molecule impacted the CRC cells might be dosage- and context-dependent. To further confirm self-renewing capacity of CEA^−/lo^ cells, we also employed organoid culture system in which human intestinal stem cells and CRC cells indefinitely self-renew and form crypt-like organoid structures [17, 18]. Under specific culture conditions, CEA^−/lo^ cells generated more organoids than CEA^+^ cells, indicating that CEA^−/lo^ cells harbor cancer stem cells (Figure 4E). Finally, we serially passaged CEA^+^ and CEA^−/lo^ cells derived from corresponding tumors. By the 2° generation, CEA^−/lo^ cells maintained relatively constant high tumorigenicity, whereas CEA^+^ cells failed to generate tumors (Figure 4F). These data clearly indicate that CEA^−/lo^ CRC cells possess higher self-renewing capacity *in vitro* and *in vivo*, enrich CSCs.

### CEA^−/lo^ CRC cells possess higher metastatic capacity

**Figure 5:**
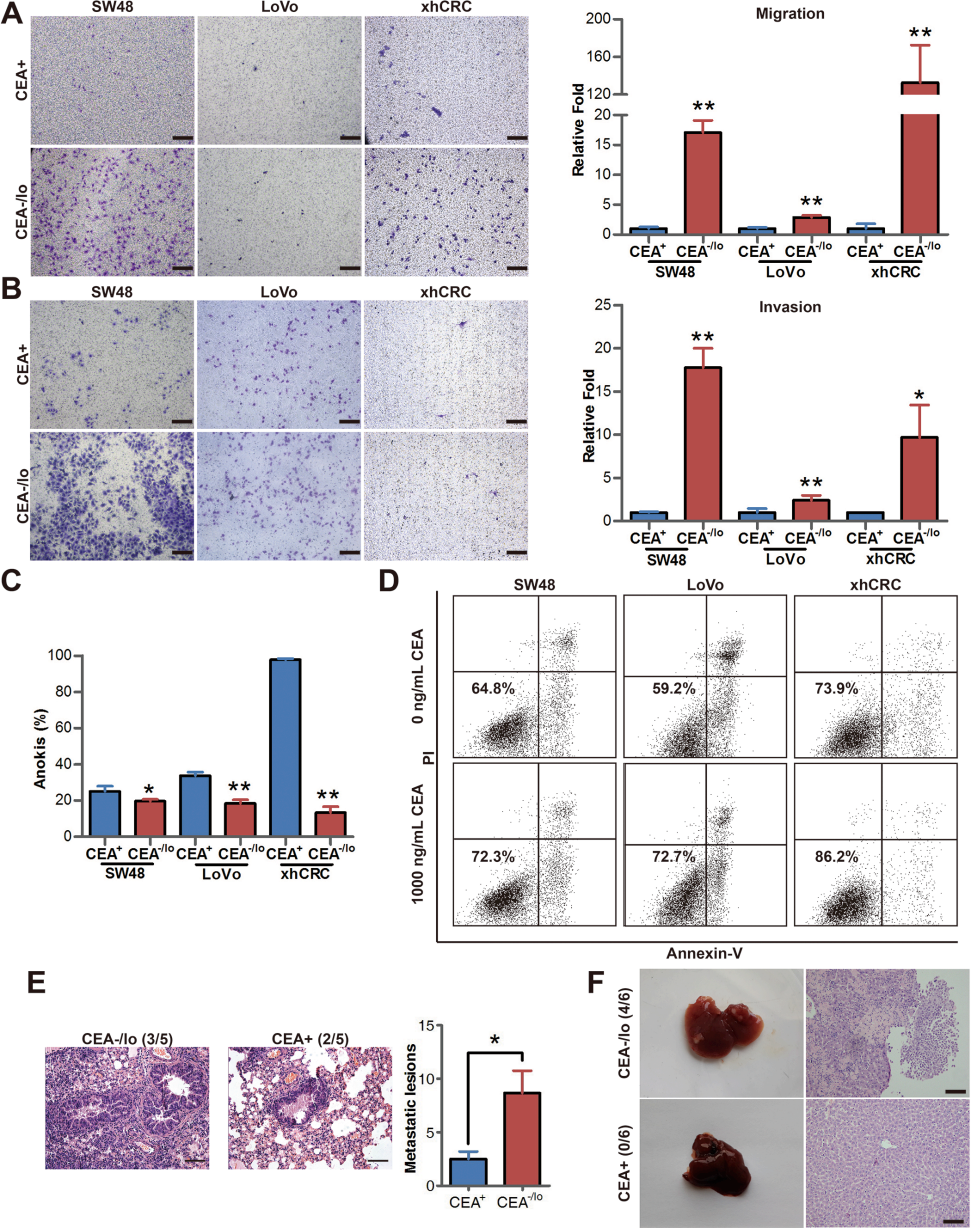
CEA^−/lo^ CRC cells possess higher migration, invasion, anti-anoikis and metastatic capacity (**A**–**B**) Transwell migration/invasion assays. CEA^+^ and CEA^−/lo^ SW48, LoVo and xhCRC cells were purified out and incubated in 37°C, after 12 (migration assays) or 24 (invasion assays) hours, invaded cells were quantified (right panel) and photographed (left panel). Mean ± SD from triple experiments. Scale bars: 100 μm. (**C**) FACS analysis of apoptosis on CEA^+^ and CEA^−/lo^ cells when underwent anoikis. Data are represented as mean ± SD from triple experiments; **P < 0.01. (**D**) FACS analysis of apoptosis on exogenous CEA treated anchorage-independent culturing CEA^−/lo^ cells. (**E**) Representative images of H&E staining of lung metastases (left panels, scale bars: 100μm) and numbers of visible metastases (right panel) in NOD/SCID mice by injecting CEA^+^ and CEA^−/lo^ cells of xhCRC to caudal veins (*n* = 5 per group). Data are presented as mean ± SD; *P < 0.05. (**F**) Representative images of liver metastases resulting from intrasplenic injection of CEA^+^ and CEA^−/lo^ SW48 cells into NOD/SCID mice (*n* = 6 per group, left panels) and H&E staining of metastases morphology (right panels). Scale bars: 100 μm.

CEA molecule was found to mediate intercellular adhesion [9], and intercellular adhesion is considered to intimately involved in tumor metastasis [19]. To explore whether CEA^−/lo^ CRC cells are intrinsically different from their corresponding CEA^+^ cells in metastatic potential, we performed transwell migration and invasion assays for purified CEA^−/lo^ and CEA^+^ CRC cells. Surprisedly, CEA^−/lo^ cells possessed significantly enhanced cell migration and invasive activity (Figure 5A–5B). Furthermore, we conducted anokis assay to access cell death of CEA^+^ and CEA^−/lo^ CRC cells when detached from surrounding extracellular matrix. Unexpectedly, CEA^+^ CRC cells, especially CEA^+^ xhCRC cells, showed an increased apoptosis (Figure 5C and Supplementary Figure S4A), suggesting that CEA^−/lo^ cells survive better in anokis assay and thus may possess higher metastatic potential. Moreover, when treated with exogenous CEA, CEA^−/lo^ cells exhibited a remarkable increased anoikis resistance during CEA treatment, while free CEA took slightly effect on CEA^+^ cells (Figure 5D and Supplementary Figure S4B–4D), suggesting that CEA molecule, secreted by CEA^+^ CRC cells, may protect CEA^−/lo^ cells from anoikis thus contributes to metastasis.

Next, we examined metastatic potential using injection of purified CEA^+^ and CEA^−/lo^ xhCRC cells into caudal veins of female NOD/SCID mice. As shown in Figure 5E, CEA^−/lo^ xhCRC cells initiated more metastases in lungs. We also conducted liver metastasis model, a widely used metastatic model in CRC research [20], by intrasplenic injecting 100,000 cells of CEA^+^ and CEA^−/lo^ SW48 cells. CEA^−/lo^ SW48 cells showed a significantly increased metastatic capacity while 100,000 CEA^+^ cells did not initiate metastatic lesions (Figure 5F). Overall, these results clearly demonstrate that CEA^−/lo^ CRC cells possess higher metastatic capacity. And serum CEA may play an important role of protecting CEA^−/lo^ cells during travelling to foreign organs.

### Distinct molecular and biological properties of CEA^−/lo^ and CEA^+^ CRC cells

**Figure 6:**
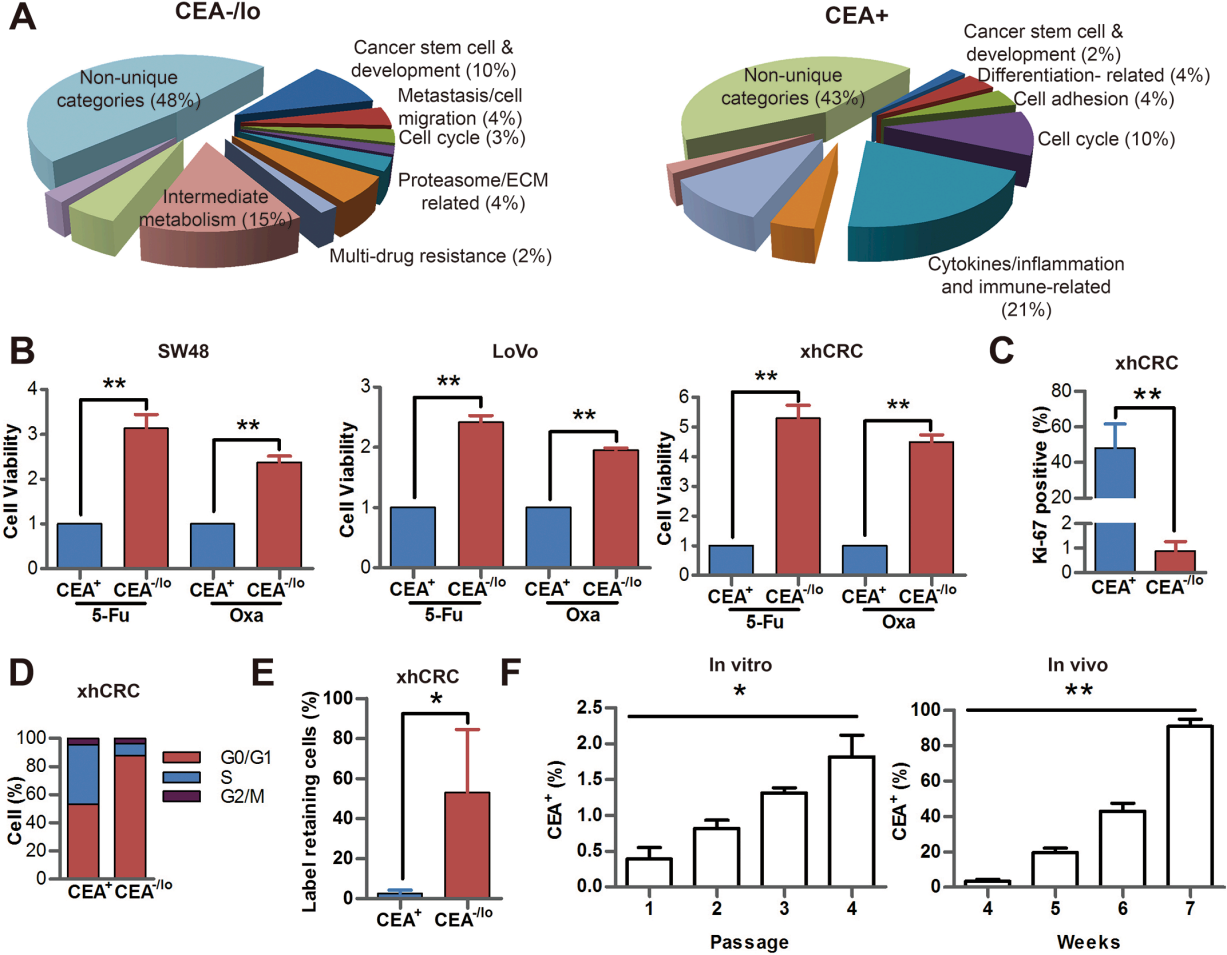
Distinct molecular and biological properties of CEA^−/lo^ and CEA^+^ CRC cells (**A**) Distinct gene expression profiles of CEA^−/lo^ and CEA^+^ xhCRC cells. Shown are pie charts of gene categories (percent indicated) overexpressed in CEA^−/lo^ cells (left) and CEA^+^ cells (right). (**B**) CCK-8 activity assays of SW48, LoVo and xhCRC CEA^+^ and CEA^−/lo^ cells upon treatment of 5-Fu or oxaliplatin for 2 days. **P < 0.01. (**C**) FACS analysis on Ki-67 expression of freshly purified CEA^+^ and CEA^−/lo^ xhCRC cells. **P < 0.01. (**D**) Cell cycle analysis in purified CEA^+^ versus CEA^−/lo^ xhCRC cells. Shown are the mean percentages of cells in different phases of the cell cycle. (**E**) Percentage of Dil-retaining cells in purified CEA^+^ versus CEA^−/lo^ xhCRC cells. Data are represented as mean ± SD from three independent experiments, *P < 0.05. (**F**) FACS analysis on CEA expression of purified CEA^−/lo^ CRC cells cultured *in vitro* and *in vivo.* For *in vitro* assays, CEA^−/lo^ xhCRC cells were cultured in DMEM with 10% FBS. *P < 0.05 (left panel). For *in vivo* assays, CEA^−/lo^ CRC cells were subcutaneously implanted into the flanks of NOD/SCID mice (right panel).

Whole genome transcriptome profiling in purified CEA^−/lo^ and CEA^+^ xhCRC cells revealed distinct gene expression patterns in two subsets (Figure 5A and Supplementary Table S3). A total of 165 genes were highly expressed, whereas 49 genes were underexpressed (fold change ≥ 2.0, *P* < 0.05). A combination of Gene Ontology analysis and literature-based curation put many of these differentially expressed genes into distinct functional categories, demonstrating that CEA^−/lo^ cells highly expressed genes related to stem cell and development and metastasis/cell migration (Figure 6A and Supplementary Table S3–S4). Indeed, the cancer stem cell-features and metastatic potential of CEA^−/lo^ cells had been already identified by *in vitro* and *in vivo* functional assays (Figures 2–4).

The CEA^−/lo^ xhCRC and LoVo cells overexpressed several ABC transporters-related genes, suggesting that CEA^−/lo^ CRC cells may display cell autonomous resistance to chemotherapy (Figure 6A and Supplementary Table S3) [21, 22]. Upon chemotherapy treatment, both CEA^+^ and CEA^−/lo^ cells showed a decrease in relative viability, but CEA^−/lo^ cells were significantly less sensitive to chemotherapeutic agents (Figure 6B).

Microarray profiling also revealed that 6% of lower expression genes in the CEA^−/lo^ xhCRC cells were related to cell-cycle progression, suggesting that CEA^−/lo^ xhCRC cells may be more quiescent than CEA^+^ cells, which may contribute to chemoresistance. Several lines of evidence supported this suggestion. First, CEA^+^ and CEA^−/lo^ xhCRC cells had 48.14% and 0.8%, respectively, of Ki-67^+^ cells (Figure 6C and Supplementary Figure S5A). Second, cell-cycle analysis revealed a larger percentage of CEA^−/lo^ cells in G0/G1 phase (Figure 6D and Supplementary Figure S5B). Third, we used DiI, a fluorescent lipophilic cationic indocarbocyanine dye, to label cell membrane, followed by implanting DiI-labeled cells into NOD/SCID mice. As cell divided, lipophilic dye that combined to membrane gradually diluted and quiescent cells remained dye positive [23, 24]. Label retaining cell experiments demonstrated that CEA^−/lo^ cells showed increased proportions in DiI^+^ fractions upon a 4-week chase, implying that CEA^−/lo^ cells are slowly cycling cells *in vivo* (Figure 6E and Supplementary Figure S5C). Interestingly, we also observed that purified CEA^−/lo^ xhCRC cells could regenerate CEA^+^ cells *in vitro* and *in vivo*. When accurately purified CEA^−/lo^ xhCRC cells were cultured in DMEM-10% FBS for several passages, percentage of CEA^+^ cells slightly increased during passaging *in vitro* (Figure 6F). We also chased CEA expression *in vivo* by implanting purified CEA^−/lo^ cells into NOD/SCID mice, and found that percentage of CEA^+^ cells significantly increased, suggesting that CEA^−/lo^ cells, during tumor progression, can regenerate CEA^+^ cells *in vivo* (Figure 6F). These data demonstrated that CEA^−/lo^ CRC cells were quiescent and resistant to chemotherapeutic agents, and could give rise to CEA^+^ cells, indicating that the CEA^−/lo^ factions are slow cycling and can differentiate into CEA^+^ cells.

### Inhibition of IGF1R targets CEA^−/lo^ cells

**Figure 7:**
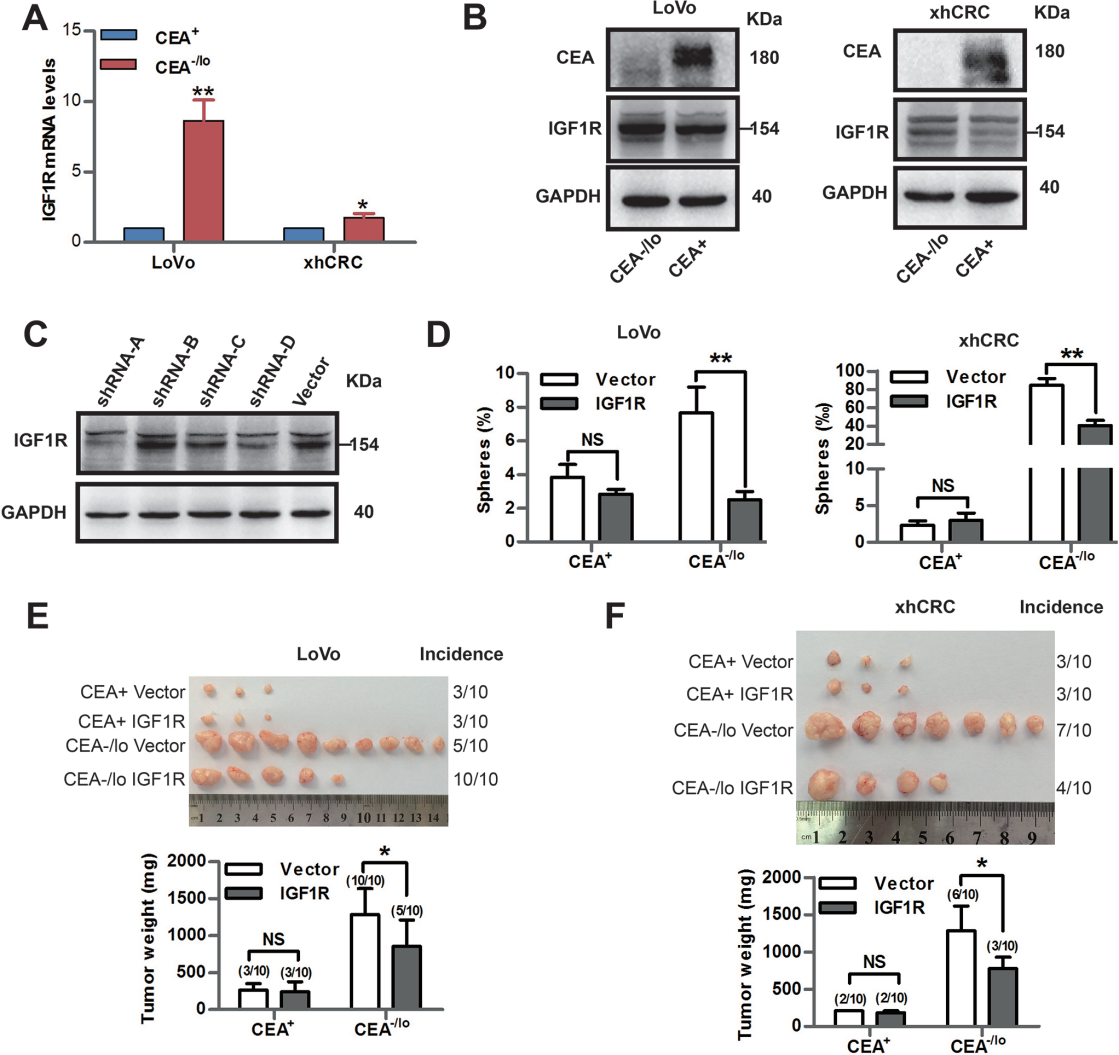
Inhibition of IGF1R targets CEA^−/lo^ cells (**A**) qPCR analysis of IGF1R mRNA levels in CEA^+^ and CEA^−/lo^ cells. *P < 0.05, **P < 0.01. (**B**) Representative immunoblot analysis of CEA and IGF1R in CEA^+^ and CEA^−/lo^ cells. Loading control was assessed by GAPDH. The 154KD IGF1R band was indicated with a short bar. (**C**) Immunoblot analysis of knocking down effect of IGF1R-shRNA on xhCRC. Loading control was assessed by GAPDH. The 154KD IGF1R band was indicated with a short bar. (**D**) Sphere-formation assays of CEA^+^ and CEA^−/lo^ cells infected by IGF1R shRNA lentivirus or vector. **P < 0.01. (**E**–**F**) Tumor transplantation assays of CEA^+^ and CEA^−/lo^ cells infected by IGF1R shRNA lentivirus or vector. Tumor images and incidences were indicated and data are represented as mean ± SD, *P < 0.05.

Expression of IGF1R is up-regulated in CSCs in human colorectal cancer [25, 26]. Therapeutic strategies targeting IGF1R were applied to clinical trials to date [27]. Therefore, we compared IGF1R expression of CEA^+^ and CEA^−/lo^ cells. Consistent with microarray data, qPCR and western blotting revealed higher levels of IGF1R mRNA and protein in CEA^−/lo^ cells while there was no significant difference in IGF1 expression between CEA^+^ and CEA^−/lo^ cells (Figure 7A, 7B and Supplementary Figure S6A–6C). We then employed lentivirus-mediate shRNA to target IGF1R. Knocking down of it was confirmed by western blotting assays (Figure 7C). Knocking down of IGF1R significantly decreased sphere-forming capacity of CEA^−/lo^ CRC cells, whereas had no effects on CEA^+^ cells (Figure 7D). And furthermore, upon overexpressing of IGF1R, CEA^+^ CRC cells possessed an increased sphere-forming capacity (Supplementary Figure S6D–6F), suggesting that IGF1 singnaling pathway may positively regulate self-renewing of CEA^+^ and CEA^−/lo^ cells. More importantly, knocking down of IGF1R reduced tumor-propagating capacity of CEA^−/lo^ CRC cells without affecting tumorigenic ability of CEA^+^ CRC cells (Figure 7E). These results suggest that IGR1R activity is functionally important in tumorigenicity of CEA^−/lo^ cells and IGR1R inhibition may be a potential therapeutic strategy to eradicate CEA^−/lo^ cells.

## DISCUSSION

CEA was first identified as a tumor associated antigen from human colon cancer tissue extracts in 1965 by Gold and Freedman [28]. Serum CEA has been applied to colorectal cancer as a tumor marker for decades [2, 29]. It was assumed that CEA, as an oncofetal antigen, expressed during fetal life, was absent in health adult tissues and re-expressed in cancer tissues [28]. In fact, in healthy individuals, serum CEA can not be detected because CEA protein is only expressed and secreted by mature columnar epithelial cells facing the free luminal surface and highly differentiated columnar epithelial cells at the crypt [6]. Early studies have demonstrated that CEA protein blocks differentiation of the cells and functions as oncogenic activity [4]. However, recent studies identified that normal adult intestinal stem cells are confined to the bottom of crypts and are negative for CEA, CEA expression increases in highly differentiated and mature columnar epithelial cells that locates near or in the surface of the villi [5, 6]. Various studies have demonstrated that intestinal cancer stem cells may originate from intestinal stem cell population upon gene mutations [5, 30, 31], implying that CEA^−/lo^ CRC cells may represent a critical source in CRC progression and metastasis.

Serum CEA is a recommended diagnostic and prognostic indicator of CRC [2]. However, early findings and our data revealed that elevated serum levels of CEA do not necessarily connote elevated tumor tissue levels of CEA [32, 33]. Elevated serum CEA levels in advanced CRC may be due to increased access of CRC cells to bloodstream and/or related to increased tumor mass in which CEA^−/lo^ cells can differentiate into CEA^+^ cells. Tissue CEA expression in CRC tumors is positively correlated with the degree of differentiation [7, 8, 34, 35]. Consistent with these clinical observations, we have performed two different analysis methods (i.e., FACS and IHC) in untreated CRC tumor tissues, both have shown that untreated CRC tumor tissues contain CEA^+^ and CEA^−/lo^ cells, and importantly, the abundance of CEA^−/lo^ CRC cells is enriched in poorly differentiated tumors or higher grade tumor areas. Strikingly, survival analysis reveals that lower tumor tissue CEA mRNA levels positively correlate with reduced patient survival.

CEA^−/lo^ and CEA^+^ CRC cells possess distinct tumor-initiating/tumor-propagating capacity, biological properties and gene expression profiles. First, using limiting dilution assays, a widely used function assay for reading out tumor-initiating cells [24, 36], it demonstrated that CEA^−/lo^ CRC cells (i.e. SW48, LoVo and xhCRC) highly enriched for tumorigenic cells. Second, serial sphere-formation assays and serial tumor transplantation assays revealed that CEA^−/lo^ cells were more clonogenic, possessed long-term clonogenicity and tumor-regenerating capacity. Third, whole genome transcriptome analysis revealed that CEA^−/lo^ cells preferentially expressed several genes including CD44, IGF1R etc., which were previously reported to associate with development and cancer stem cell functions [25, 26, 37]. Consistent with the previous studies [25, 38], we also demonstrated that, using CRC cell line and xenograft tumor cells, IGF1R positively mediated sphere-forming capacity, tumor-initiating/propagating capacity of CEA^−/lo^ cells but not CEA^+^ cells. Fourth, our analysis has shown that CEA^−/lo^ cells can regenerate CEA^+^ daughter cells *in vitro* and *in vivo*. One recent study has also demonstrated that SOX9, which was highly expressed around the bottom of the crypts and regulated cancer stemness, inhibited differentiation of CRC cells and carcinoembryonic antigen gene, suggesting that CEA expression was downregulated in CSCs [39]. Taken together, the biological, molecular and tumorigenic properties of CEA^−/lo^ cells presented herein, coupled with location of CEA^−/lo^ cell in normal adult colonic crypts and origin of cancer cells [6, 30, 31, 39], suggesting that CEA^−/lo^ CRC cell population, harboring self-renewing CSCs, may represent a critical source in maintaining colorectal cancer [37, 40].

The multistep process of invasion and metastasis has been schematized as a sequence of discrete steps, which includes transit of cancer cells through circulation systems and colonization in distant tissues, often termed the invasion–metastasis cascade [12]. CSCs have previously been reported to mediate invasion-metastasis cascade [13]. Indeed, our data revealed that CEA^−/lo^ cells possessed higher cell migration and invasion capacity than CEA^+^ CRC cells. Furthermore, when compared with CEA^+^ cells, CEA^−/lo^ CRC cells (i.e., SW48, LoVo and xhCRC cells) were more resistant to anoikis, a critical property of anchorage-independent survival [41]. Most significantly, we illustrated that CEA^−/lo^ cells initiated more metastases when employing either intrasplenic injection or caudal vein injection models in NOD/SCID mice. Of note, CEA functions as intercellular adhesion molecule thus mediating homotypic aggregation [6, 9], and furthermore, forced expression of CEA promotes homotypic aggregation thus inhibiting anoikis and then enhances metastasis [10, 42]. It may be interpreted that CEA molecule and CEA^−/lo^ CRC cells differently function in mediating metastasis. Indeed, exogenous CEA-treated CEA^−/lo^ cells exhibited a significant decrease in anoikis, implying that CEA molecule may partially take effects through protecting CEA^−/lo^ cells from anoikis hereby contributes to metastasis. In addition, CEA^−/lo^ CRC cells, since highly enriched for CSCs, were more easily to form metastatic lesions when compared to CEA^+^ CRC cells. Circulation tumor cells (CTCs) have been emerged as a potential biomarker in the diagnosis and prognosis in colorectal cancer [43]. However, investigations on CEA^+/−^ CTCs in CRC are limited. Serum CEA and CTCs both predicted poor prognosis, although there appeared to be no correlation between the CTCs and serum CEA values [44]. A few studies have claimed that CEA expression in CTCs may serve as a predictor of bad prognosis in CRC [43, 45], In contrast to these studies, other studies demonstrated that CEA mRNA in blood was not considered to be an independent prognostic factor in CRC [46–48]. One interesting study, in which only 6% CRC patients showed evaluated CEA mRNA in CTCs while 38% CRC patients showed at least 100-fold increased CK20 using real time quantified PCR, suggested that CEA expression on CTCs may be negative or of low level, implying that CEA^−/lo^ CTCs may play an important role in metastasis [49]. Together with the fact that serum CEA is a predictor of bad prognosis, CEA^−/lo^ CTCs associated with high CEA circulating level may be a bad prognostic.

It has been reported that CSCs mediate chemotherapy resistance in a variety of tumors [50, 51]. In the present study, CEA^−/lo^ CRC cells are more quiescent manifesting as increased proportion in DiI- retaining cells, larger percent of cells in G0/G1 phase and lower expression of Ki-67 *in vivo*, implying that CEA^−/lo^ cells may be more resistant to chemotherapy [52]. Indeed, CEA^−/lo^ CRC cells are more resistant to chemotherapeutic agents. Besides that, microarray data reveals that CEA^−/lo^ cells overexpress several genes such as ABCC5, ABCG1 and ABCB10, which involve in drug efflux [21, 22], suggesting that CEA^−/lo^ cells may contribute to chemoresistance via overexpressing drug efflux related genes.

We have provided evidence that CEA^−/lo^ CRC cells, which preexist in the tumors, are molecularly and functionally distinct from CEA^+^ cells and positively correlate with tumor grade and poor prognosis. CEA^−/lo^ cells harbor self-renewing tumor-initiating cells and can generate CEA^+^ progeny. Furthermore, CEA^−/lo^ cells that display properties of cell mobility and anoikis resistance are responsible for cancer metastasis. Inhibition of IGF1R may be a potential therapeutic strategy of CEA^−/lo^ cells. However, more work addressing the mechanism that cellular CEA regulates cell differentiation should be done and novel therapeutic targeting CEA^−/lo^ cells should be developed and used in conjunction with conventional chemotherapy in order to eradicate all CRC cells and prevent recurrence and metastasis.

## MATERIALS AND METHODS

### Cells and antibodies

Human colon cancer cells, LoVo and SW48, were purchased from ATCC (Manassas,VA) and cultured in DMEM media (Invitrogen, CA, USA) supplemented with 10% FBS (Gibco, NY, USA) in a 37°C humidified incubator with an atmosphere of 5% CO_2_ and 95% air.

The antibodies used in the present study included: mouse anti human CEA (clone: Ab-3; Thermo Scientific, MA, USA), anti-biotin microbeads (Miltenyi Biotec, CA, USA), mouse anti human Epcam-APC (clone: HEA-125; Miltenyi Biotec, CA, USA), mouse anti human CD44 (clone: 156-3C11, Cell Signal Technology, USA), mouse anti-biotin FITC (clone: Bio3-18E7; Miltenyi Biotec, CA, USA), Alexa Flour 488 conjugated to goat anti mouse IgG (Jackson ImmunoResearch Laboratories, PA, USA), rabbit anti human cytokeratin 20 (clone: EPR1622Y; Abcam, CA, USA), mouse anti human GAPDH (Santa Cruz Biotechnology, CA, USA), rabbit anti human IGF1R (1159–1163, Abcam, CA, USA), goat anti human IGF-1 (Abcam, CA, USA).

### Tissue collection and isolation of cancer cells

Human colorectal adenocarcinoma samples were obtained under IRB-approved guidelines and with informed patient consent at Tongji Hospital of Huazhong University of Science and Technology, Wuhan, China. Fresh specimens were minced into small pieces with scissors. Completely minced pieces were then incubated in serum free DMEM/F12 medium (Life technologies, NY, USA) containing 1.5mg/ml collagenase IV (Gibco, NY, USA), 20 ug/ml hyaluronidase (Sigma-Aldrich, MO, USA), 1% penicillin/streptomycin (Life technologies, NY, USA) at 37°C for 1 to 2 hours. The specimens were mechanically dissociated every 15 minutes by pipetting with a 15-ml pipette. At the end of dissociation, cells were filtered through a 40-μm nylon mesh, washed with PBS. Red blood cells were then eliminated using red blood cells lysis buffer (Biolegend, CA, USA). Single cells were washed with PBS twice and resuspended in PBS.

### Establishment of CRC xenograft (xhCRC)

Human CRC sample was obtained from a female patient with Dukes' C stage and tumor grade 2 adenocarcinoma [36]. Xenograft tumors were established as described [53].

### Immunohistochemistry

Immunohistochemistry of formalin-fixed paraffin-embedded human CRC sections was performed as described [24]. Five fields were chosen from each slide by two experienced pathologists. CEA levels were evaluated according to immunoreactive score (IRS): IRS = SI (staining intensity) × PP (percentage of positive cells). SI was determined as 0 is negative; 1, weak; 2, moderate; and 3, strong. PP was defined as 0 is negative; 1, ≤ 10% positive cells; 11–50% positive cells; 51–80% positive cells; and 4, more than 80% positive cells [54].

### Immunofluorescence microscope

Cells were fixed and immunostained as described previously [36]. The following antibodies were used to detect antigens: CEA (1:100; Thermo Scientific, UK), EpCAM (1:100, Miltenyi Biotech), cytokeratin 20 (1:100; Dako, Denmark). Nuclei were stained with DAPI (4',6-Diamidino-2-phenylindole, Sigma). Details were described in supplementary methods.

### Correlating CEA mRNA levels with patient overall survival

*SurvExpress*, an online biomarker validation tool, was utilized to perform survival analysis [14]. The colon metabase including GSE12945, GSE14333, GSE17536, GSE17537, GSE31595 and GSE41258 was chosen and survival profiles were compared based on high and low CEACAM5 mRNA expression, and censored for survival in months.

### Cell sorting

Cells were labeled with CEA antibodies. CEA^+^ and CEA^−/lo^ cells were purified using magnetic cell separation (MACS) or fluorescence-activated cell sorting (FACS). Details were described in supplementary methods.

### Animal studies

4 to 6-week-old female BALB/c-nu mice and NOD/SCID mice were purchased from Beijing HFK Bioscience CO., LTD. (Beijing, China) and maintained according to institutional guidelines of the Huazhong University of Science and Technology Animal Care committee. To generate tumors, cells were suspended in PBS/Matrigel (BD Biosciences, CA, USA) mixture (1:1 volume) and injected subcutaneous tissue of the flanks using 27-gauge needles. To establish metastasis models, cells were suspended in PBS and injected into the spleens or the caudal veins using 29-gauge needles. Details were described in supplementary methods.

### Clonal culture, sphere-formation and organoid culture

Basic procedures for clonal culture, sphere-formation assays and organoid culture were previously described [24, 55]. For clonal culture, purified CEA^+^ and CEA^−/lo^ cells were plated in a six-well culture plate at a density of 100, 200 or 300 cells/well. Clones with ≥ 50 cells were scored ~2 weeks after plating. For sphere-formation assays in CRC cell lines, xenografts, purified CEA^+^ and CEA^−/lo^ cells were plated at 200 cells/well (SW48 and LoVo cells) or 1,000 cells/well (xenografts) in ultra-low attachment (ULA) plates. Spheres that raised within 1–2 weeks and ≥ 50μm were presented as clonogenicity (% or ‰). For organoid culture, purified CEA^+^ and CEA^−/lo^ cells were plated at 100 cells/well or single cell per well and cultured under special conditions. Details were described in supplementary methods.

### Transwell migration and invasion assays

Purified CEA^+^ and CEA^−/lo^ cells were resuspended in 100 μL serum free DMEM medium and seeded on the upper chamber of transwell 24-well plates (8 μm pores; Corning, NY, USA). Cells were allowed to migrate for 12 hours or invade for 24 hours. Invaded cells were stained with 1% crystal violet solution and images were captured using an inverted microscope (CKX41, Olympus). For each chamber, ten fields were chosen and stained cells were counted.

### Anoikis and FACS analysis

Cell anoikis was induced by anchorage independent culture as described [56]. Cells underwent anoikis were analyzed by FACS using an Annexin-V-FITC Apoptosis Detection Kit (KeyGEN Biotech, Nanjing, China). Details were described in supplementary methods.

### cDNA microarray and analysis

Total RNA was extracted from purified CEA^+^ and CEA^−/lo^ cells and applied to human Affymetrix GeneChip arrays (Affymetrix, CA, USA) in CapitalBio Corporation (Beijing, China). Data analysis was performed using Microarray Suite version 5.0. Genes were considered differential expressed if the fold change was greater than 2.0 fold in either direction and the *P*-value was less than 0.05. These genes were analyzed by gene ontology (GO) analysis and sorted into categories based on GO analysis and exhaustive literature search (i.e., manual curation) [24]. Microarray data has been deposited in the NCBI GEO database under the accession number GSE72398.

### Quantitative RT-PCR and western blot analysis

Total RNA and protein isolation were described in supplementary methods. A detailed description of the methods and primers were provided in the Supplement.

### *In vitro* treatment with 5-Fu or oxaliplatin

Cell death analysis of 5-Fu or Oxa treated CEA^+^ and CEA^−/lo^ cells were measured using Cell Counting Kit-8 (Dojindo, Japan). Briefly, cells were seeded in 96-well plates at 3,000 cells per well. After 12 hours post plating, the cells were treated with either 5-Fu (1 μM; Sigma, MO, USA) or oxaliplatin (1 μM; Sigma, MO, USA) for 72 hours. Then, 10 μl CCK-8 solution was added to each well and the plates were incubated at 37°C for 1 hour. Finally, relative cell viability was measured using a microplate reader at 450 nm.

### Label retaining cell experiments using DiI dye-retention assays

Single xhCRC cells were obtained from xenograft tumors as described above. Before stained with DiI (Santa Cruz Biotechnology, CA, USA), the proportions of CEA^+^ and CEA^−/lo^ cells were measured by FACS. And then 1 × 10^6^ DiI-labeled xhCRC bulk cells were injected into the flank of NOD/SCID mice. When Dil-labeled cell-derived tumors grew up, the tumors were dissociated into single cells, followed by evaluating DiI^+^ cells using FACS.

### Chasing CEA expression of cultured CEA-/lo cells

For chasing CEA expression of cultured CEA^−/lo^ cells *in vitro*, 1 × 10^6^ purified CEA^−/lo^ cells were plated in 10-cm culture dish. During each passage of xhCRC CEA^−/lo^ cells, the expression of CEA was evaluated by FACS.

For chasing CEA expression of cultured CEA^−/lo^ cells *in vivo*, 1 × 10^6^ purified CEA^−/lo^ cells were implanted into the flank of NOD/SCID mice. When tumors grew up, the tumors were dissociated into single cells and CEA expression was measure by FACS.

### Knockdown or overexpress of IGF1R with lentivirus vectors

To knock down or up-regulate IGF1R expression in both cell lines and xenografts, we purchased IGF1R-shRNA lentivirus and IGF1R-overexpressing lentivirus from Shanghai SBO Medical Biotechnology (Shanghai, China). Cells were infected with IGF1R-shRNA lentivirus/ IGF1R-overexpressing lentivirus or vector for 72 hours, all at MOI of 25. The lentivirus-mediated effect on IGF1R was confirmed by Western blot analysis.

### Statistics analysis

Data are expressed as mean ± SD, unpaired two-tail Student's *t*-test or two-way ANOVA test was performed on IBM SPSS Statistics 18 to compare differences. Fisher's exact test was utilized to compare the differences between categorical data. Long-Rank test was employed to analyze the survival differences. In these analyses, statistically significant difference was defined as *P* < 0.05.

## SUPPLEMENTARY MATERIALS


