# Supplementary material for: The CEA−/lo colorectal cancer cell population harbors cancer stem cells and metastatic cells
Source: Oncotarget. 2016 Nov 2;7(49):80700–15. doi: 10.18632/oncotarget.13029 (PMC5348349; doi:10.18632/oncotarget.13029)
Supplement: Supplementary file 1 [file oncotarget-07-80700-s001.pdf]

The CEA$^{-/lo}$ colorectal cancer cell population harbors cancer stem cells and metastatic cells

Supplementary Materials

Flow cytometric analysis

To identify CEA^{+} and CEA$^{-/lo}$ cells in CRC specimens or xengrafts, tissues were processed into single cell suspension as described above. Cells were stained with biotinylated CEA antibodies (10 µl/10^{7}cells) at 4°C for 15min, followed by stained with anti-biotin FITC secondary antibodies (10 µl/10^{7}cells), EpCAM-APC (10 µl/10^{7}cells) and propidium iodide (50 µg/mL; Sigma, MO, USA) at 4°C for 15 min. Samples were analyzed using BD FACS Aria II (BD Biosciences, CA, USA).

Cell-cycle analysis was performed for purified CEA^{+} and CEA$^{-/lo}$ xhCRC cells, fixed in ice-cold 70% ethanol at 4°C overnight. Cells were then washed with PBS and incubated with PBS containing propidium iodide (50ug/ml; Sigma, USA) and RNase (10ug/ml; Sigma, USA) at room temperature for 30 minutes. Samples were analyzed using BD FACS Aria II.

Ki-67 expression was performed for purified CEA^{+} and CEA$^{-/lo}$ xhCRC cells, fixed in ice-cold 70% ethanol at −20°C for 1 hour. Cells were then washed with PBS and incubated with anti-Ki-67 antibodies (10 µl/10^{7}cells; Miltenyi Biotech) at room temperature for 30 minutes. After washed with PBS twice, cells were analyzed using BD FACS Aria II.

Magnetic sorting and fluorescence-activated cell sorting

Magnetic cell separation was performed on LoVo and SW48 cells using biotinylated CEA antibodies (Thermo Scientific, MA, USA) and anti-biotin microbeads. Quality of sorting of both CEA^{+} and CEA$^{-/lo}$ fractions was verified by flow cytometry and/or immunofluorescence staining with mouse anti human CEA monoclonal antibodies, followed by labeled with PE-conjugated goat anti mouse antibodies (Dako, Denmark).

Fluorescence-activated cell sorting was performed on xenograft tumors. To segregate CEA^{+} and CEA$^{-/lo}$ epithelial cells, immunostaining for CEA in xhCRC was described as above, and PI^{-}CEA^{+}EpCAM^{+} and PI^{-}CEA^{+}EpCAM^{+} cells were purified out.

Immunofluorescence microscopy

Immunofluorescence was performed on cytospins as described [1]. The following antibodies were used: CEA (1:100; Thermo Scientific, UK), EpCAM (1:100, Miltenyi Biotech), cytokeratin 20 (1:100; Dako, Denmark). Briefly, cells were plated on the glass coverslips overnight. Cells were then fixed by 4% paraformaldehyde (PFA) for 10 min at room temperature. After blocking with 5% (w/v) bovine serum albumin in PBS, cell were incubated with primary antibodies at 4°C overnight, followed by staining with secondary antibodies conjugated to biotin (Miltenyi Biotech) or Alexa Flour 488 (Jackson ImmunoResearch Laboratories, PA, USA) at room temperature for 2 hours. Finally, nuclei were stained with DAPI (4',6-Diamidino-2-phenylindole, Sigma) for 10min at room temperature. Cells were visualized by using a fluorescence microscope (TRRFM, Olympus) or a confocal microscope (FV1000, Olympus).

In vivo limiting dilution assays and serial tumor transplatation in NOD/SCID mice

For limiting dilution assays, varying number of CEA^{+} and CEA$^{-/lo}$ cells from different CRC cell lines or xenograft tumors were implanted into the back of Balb/c-nu mice or NOD/SCID mice (see Table 2 for the injected cell numbers). The frequency of tumor-initiating cells and statistical significance were examined using the Extreme Limiting Dilution Analysis (ELDA) software at the application website http://bioinf.wehi.edu.au/software/elda/index.html.

For serial tumor transplantation in NOD/SCID mice, CEA^{+} and CEA$^{-/lo}$ cells were purified from xhCRC and 10,000 (+10K or −10K) cells each were implanted subcutaneously in female NOD/SCID mice (n = 5 per group). The first-generation (1°) tumors were harvested 40 days later. CEA^{+} cells were purified from one tumor originally derived from purified CEA^{+} cells whereas CEA$^{-/lo}$ cells were from one tumor derived initially from purified CEA$^{-/lo}$ cells. And then, 10,000 cells each were implanted subcutaneously to generate secondary (2°) tumors in NOD/SCID mice.

Clonal culture, sphere-formation assays and organoid cultures

Basic procedures for clonal culture and sphere-formation assays were previously described [2, 3]. For clonal culture, purified CEA^{+} and $CEA^{-/lo}$ cells were plated in a six-well culture plate at a density of 100, 200 or 300 cells/well. Clones with ≥ 50 cells were scored ~2 weeks after plating. The results were expressed as cloning efficiency (%). For sphere-formation assays in CRC cell lines, xenografts, purified CEA^{+} and $CEA^{-/lo}$ cells were plated at 200 cells/well (SW48 and LoVo cells) or 1,000 cells/well (xenografts) in ultra-low attachment (ULA) plates. Spheres that raised within 1-2 weeks and ≥ 50μm were presented as clonogenicity (% or ‰). For serial sphere-formation assays, the first-generation spheres were harvested, disaggregated with 0.025% trypsin/EDTA, triturated with a 27-G needle, filtered through a 40-μm mesh, and replated as above. This process was repeated for up to 3 generations. When cells were treated with exogenous CEA, different doses of recombinant human CEA protein (R&D systems) were added every 3 days. For organoid cultures, the Matrigel (GFR, phenol free, BD bioscience, USA) were first polymerized for 10 minutes at 37°C, and 250 μL/well basal culture medium (DMEM/F12 culture medium, 10 mmol/L HEPES, Glutamax, 1 × N2, 1 × B27 [Invitrogen, USA], and 1 mmol/L N-acetylcysteine [Sigma, USA]) was overlaid containing the following growth factor combinations: gastrin (10nM, Sigma, USA) + EGF (50 ng/mL, Invitrogen, USA) +. Y-27632 (10 μmol/L, Sigma, USA) was included in the medium for the first 2 days to avoid anoikis

Anoikis and FACS analysis

Anoikis was induced as described [4]. Briefly, purified CEA^{+} and $CEA^{-/lo}$ cells were seeded at a density of 20,000 cells per well (500 μL) in a 24-well ultra-low attachment culture plate (Corning, USA). After 4 days, cells were harvested and resuspended in 500 μL binding buffer supplemented with 5 μL Annexin-V-FITC and 5 μL propidium iodide according to the instruction of Annexin-V-FITC Apoptosis Detection Kit (KeyGEN Biotech, Nanjing, China). The percentage of cells that underwent anokis was defined as the Annexin-V or propidium iodide positive population analyzed on BD FACS Aria II.

To explore the effects of exogenous CEA on CEA^{+} and $CEA^{-/lo}$ CRC cells, cells were purified and plated as described above and recombinant human CEA protein (1000 ng/mL; R&D systems) was added every 3 days. After 6 days, cells were harvested and analyzed by flow cytometry as described above.

Experimental liver and lung metastasis model for colorectal cancer

Liver metastases were generated by injecting 100,000 purified SW48 CEA^{+} and $CEA^{-/lo}$ cells into spleens of NOD/SCID mice. After 8 weeks, livers were removed and examined for metastatic lesions.

Lung metastasis was performed by injecting 1,000,000 purified xhCRC CEA^{+} and $CEA^{-/lo}$ cells through tail veins of NOD/SCID mice. After 8 weeks, lungs were removed and examined for metastatic lesions.

Quantitative RT-PCR and western blot analysis

Total RNA was extracted using RNAiso Plus (Takara, Japan) and then complementary DNA (cDNA) was synthesized using the Mixima First Strand cDNA Synthesis Kit (Thermo Scientific, MA, USA). Quantitative RT-PCR analysis was carried out using an ABI PRISM 7300 Sequence Detection System instrument with Maxima SYBR Green/ROX qPCR Master Mix (Thermo Scientific, MA, USA) according to the manufacturer's instructions. Glyceraldehyde-3-phosphate dehydrogenase (GAPDH) was used as an internal control. The details of primers are shown below: GAPDH forward: 5′-TCGTGGAAGGACTCATGACC-3′, GAPDH reverse 5′-TCCACCACCCTGTTGCTGTA-3′, CD44 forward 5′-AGCAACCAAGAGGCAAGAAA-3′, CD44 reverse 5′-GTGTGGTTGAAATGGTGCTG-3′, IGF1R forward 5′-TCGACATCCGCAACGACTATC-3′, IGF1R reverse 5′-CCAGGGCGTAGTTGTAGAAGAG-3′.

Purified cells were lysed in NP40 protein lysis buffer containing a protease inhibitor mixture (Sigma, MO, USA) and separated by sodium dodecyl polyacrylamide gel electrophoresis (SDS-PAGE) and transferred onto nitrocellulose membranes. After blocking with 5% BSA in 0.01 M TBS (pH 7.5) containing 0.5% Tween 20 (TBST), the membranes were incubated with the appropriate primary antibodies at 4°C overnight, followed by incubated with horseradish peroxidase-conjugated goat anti-rabbit-IgG or goat anti-mouse IgG secondary antibodies for 2 hours at room temperature. Finally, the protein bands were visualized using SuperSignal West Femto Maximum Sensitivity Substrate (Thermo Scientific, MA, USA).

REFERENCES

1. Patrawala L, Calhoun T, Schneider-Broussard R, Zhou J, Claypool K, Tang D. Side population is enriched in tumorigenic, stem-like cancer cells, whereas ABCG2+ and ABCG2- cancer cells are similarly tumorigenic. Cancer research. 2005; 65:6207–6219.
2. Jeter CR, Badeaux M, Choy G, Chandra D, Patrawala L, Liu C, Calhoun-Davis T, Zaehres H, Daley GQ and Tang DG. Functional evidence that the self-renewal gene NANOG regulates human tumor development. Stem cells. 2009; 27:993–1005.
3. Sato T, Stange DE, Ferrante M, Vries RGJ, van Es JH, van den Brink S, van Houdt WJ, Pronk A, van Gorp J, Siersema PD, Clevers H. Long-term Expansion of Epithelial Organoids From Human Colon,

Adenoma, Adenocarcinoma, and Barrett's Epithelium. Gastroenterology. 2011; 141:1762–1772.

4. Schackmann RC, Klarenbeek S, Vlug EJ, Stelloo S, van Amersfoort M, Tenhagen M, Braumuller TM, Vermeulen JF, van der Groep P, Peeters T, van der Wall E, van Diest PJ, Jonkers J, Derksen PW. Loss of p120-catenin induces metastatic progression of breast cancer by inducing anoikis resistance and augmenting growth factor receptor signaling. Cancer research. 2013; 73:4937–4949.

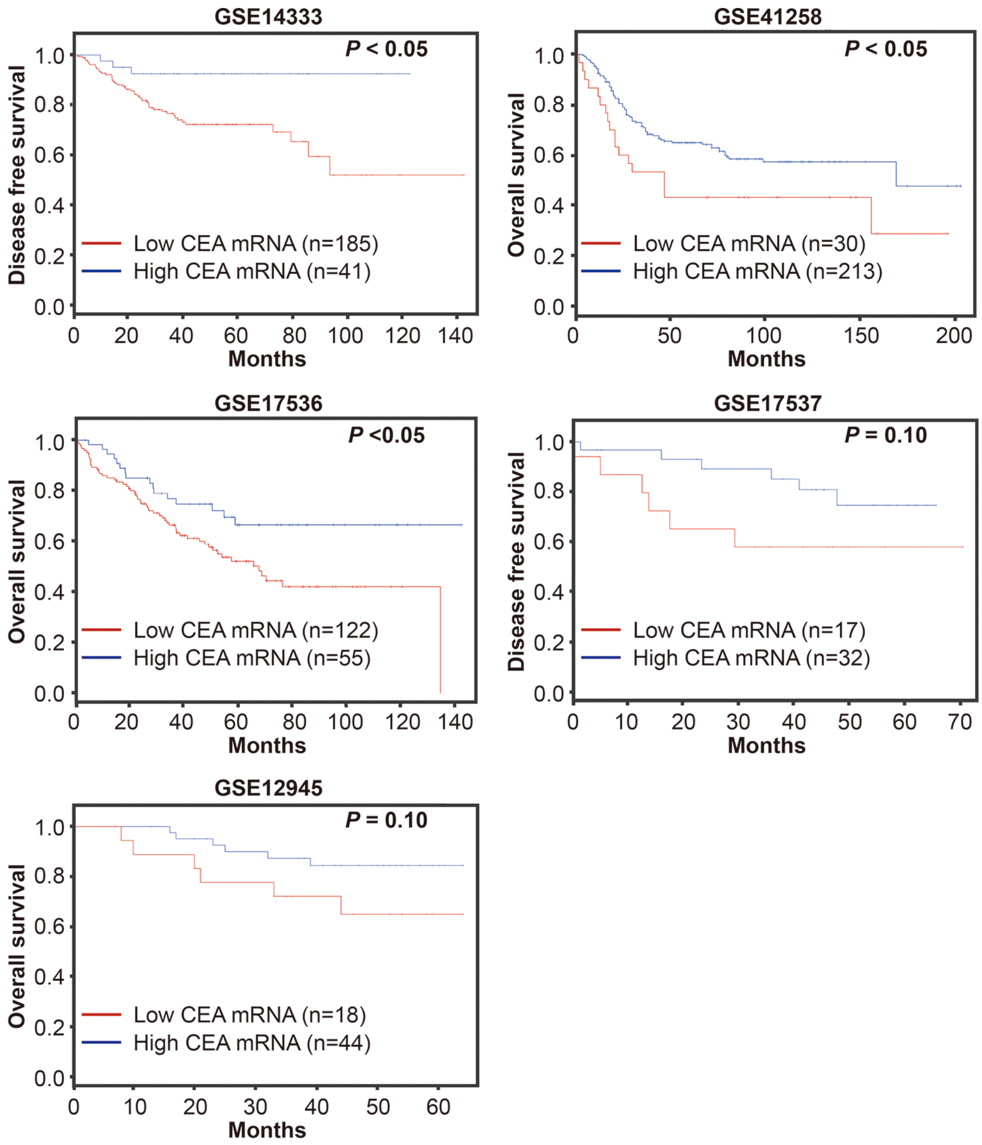

Supplementary Figure S1: Decreased tumor CEA mRNA levels positively correlate with poor prognosis. Modified graphical output from several GSE datasets (GSE14333, GSE41258, GSE17536, GSE17537 and GSE12945) included in the colon metabase using the *Survexpress* website. Every GSE dataset reveals that patients with lower CEA mRNA level have longer survival.

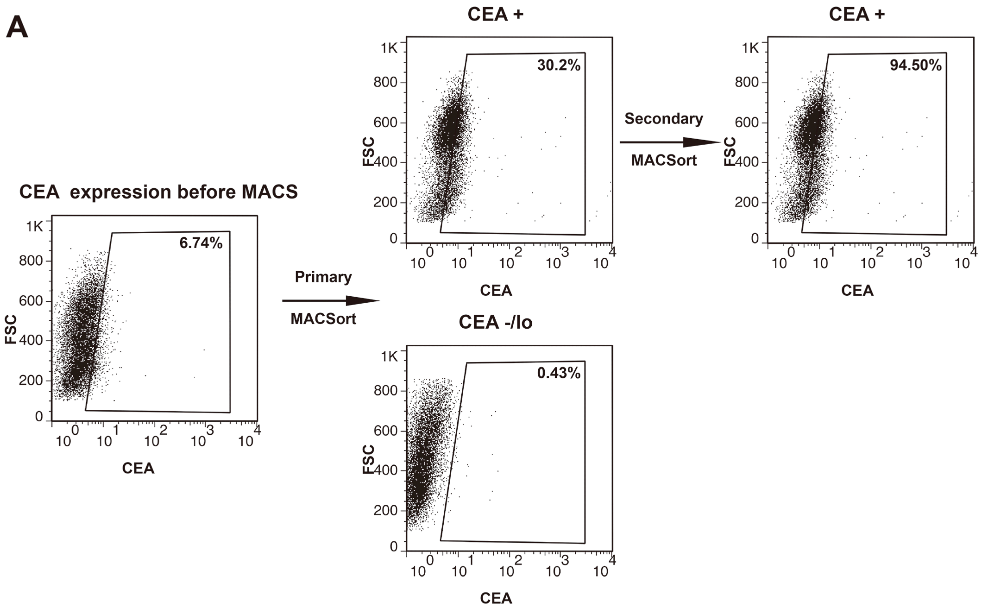

Supplementary Figure S2: Magnetic activated cell sorting of CEA$^+$ and CEA$^{-/lo}$ cells. An example of post-sorting analysis of the magnetic activated cell sorted (MACS) CEA$^+$ and CEA$^{-/lo}$ SW48 cells. In the example, in order to enrich high purity CEA$^+$ cells, micorbeads labeled cells were applied onto the MACS separation columns twice.

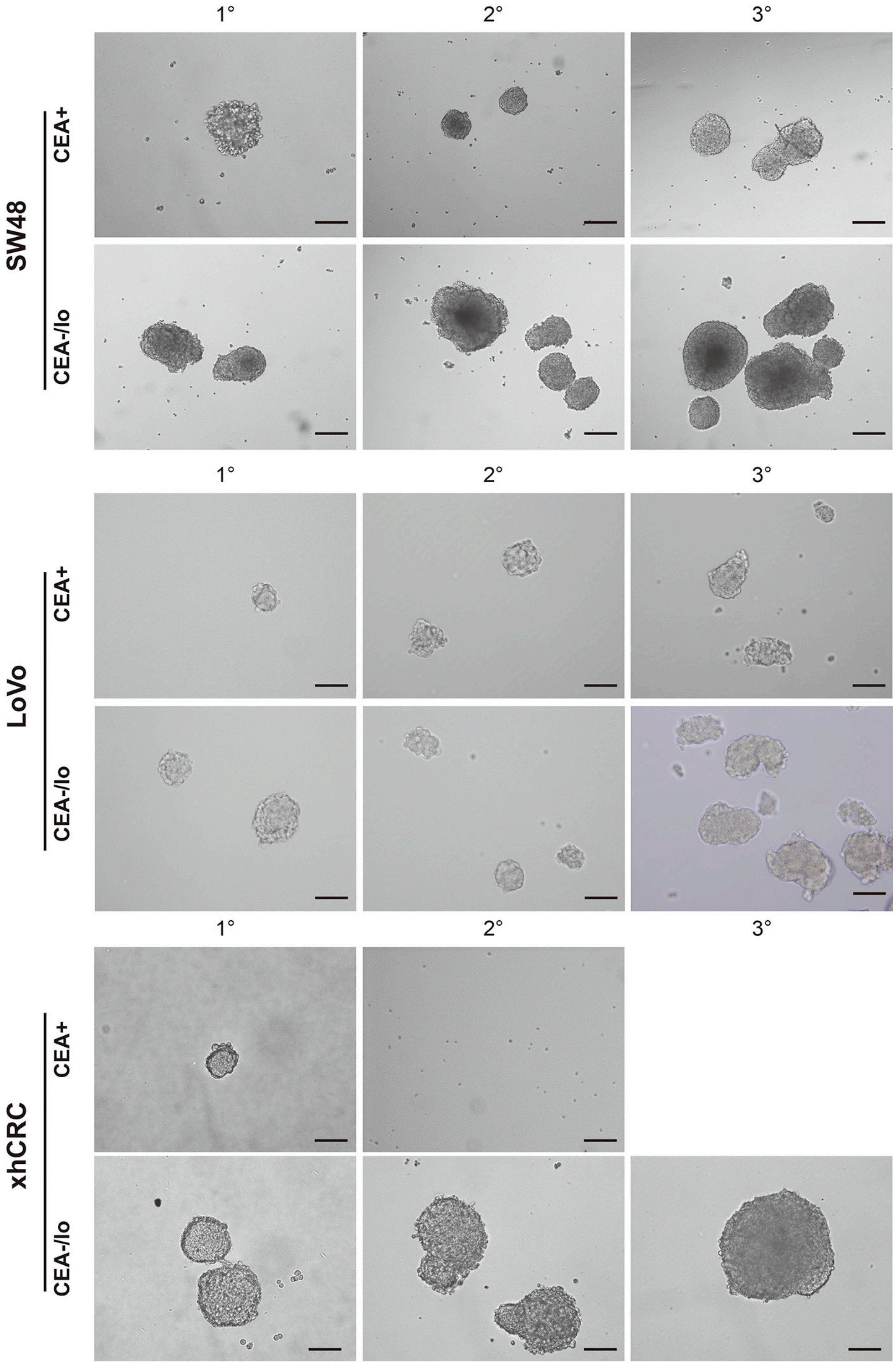

Supplementary Figure S3: CEA$^{-/lo}$ cells possessed long-term sphere-forming capacity. CEA^{+} and CEA$^{-/lo}$ CRC cells (i.e., SW48, LoVo, xhCRC) were acutely purified, and then seeded in ultra-low attachment wells. Primary spheres were formed at 1~2 weeks after plating. Note that CEA$^{-/lo}$ cells formed more spheres than corresponding CEA^{+} cells during subsequent serial passaging. And more significantly, CEA^{+} xhCRC lost sphere-propagating capacity by 2° generation, whereas CEA$^{-/lo}$ CRC cells gradually increased in the sphere-propagating capacity. Scale bars: 100 μm.

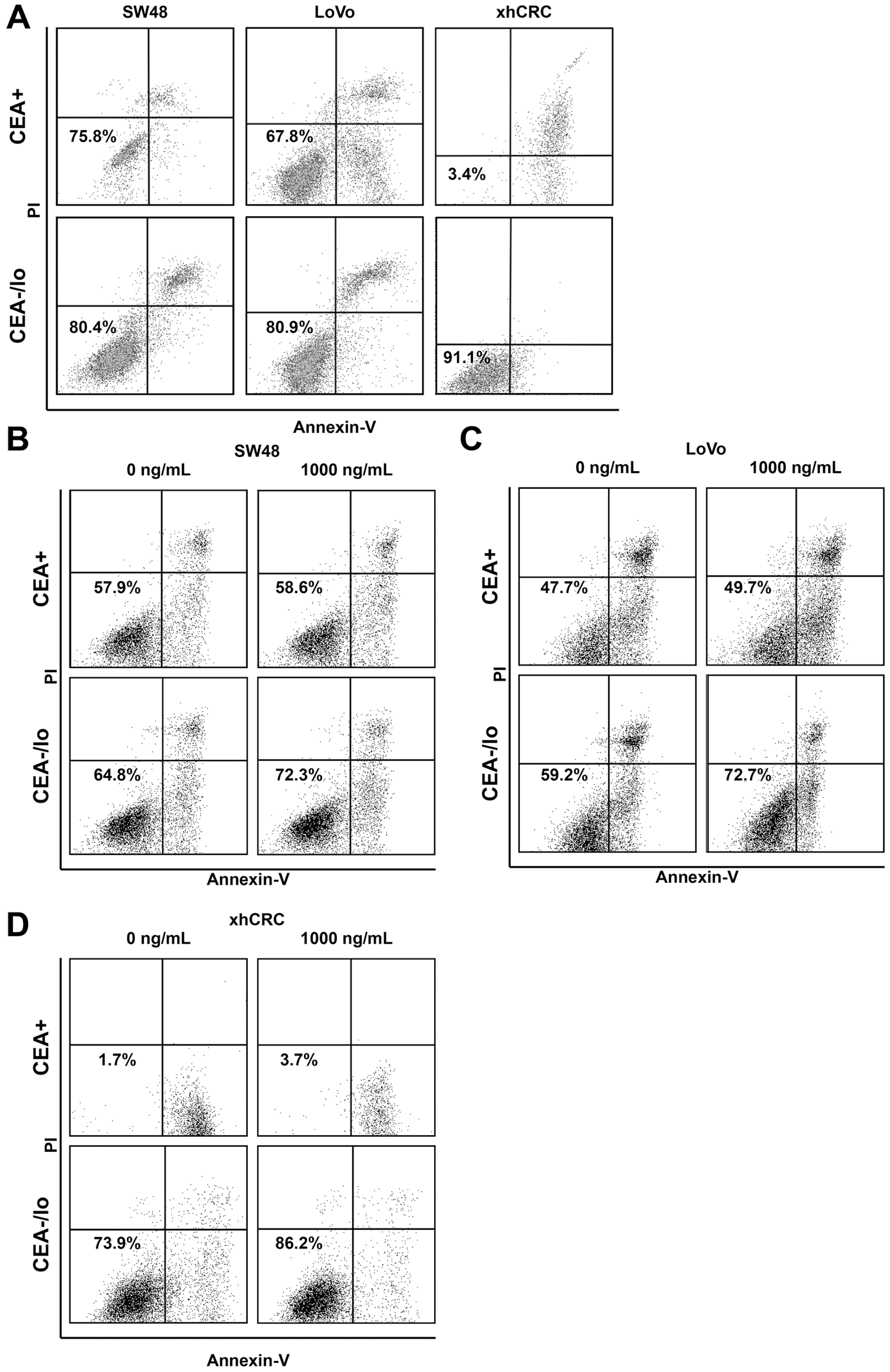

Supplementary Figure S4: CEA$^{-/lo}$ cells exhibit higher anoikis resistance capacity, and CEA molecule protects CEA$^{-/lo}$ cells from anoikis. (**A**) Single CEA^{+} and CEA$^{-/lo}$ CRC cells (i.e., SW48, LoVo, xhCRC) were acutely purified, and then seeded at a density of 20,000 cells/well in ultra-low attachment 24-wells. After 4 days anchorage-independent culture, cell apoptosis was evaluated by flow cytometry. The percentage of cells that resistant to anokis was defined as the Annexin-V and propidium iodide negative population. (**B**–**D**) Single purified CEA^{+} and CEA$^{-/lo}$ CRC cells (i.e., SW48, LoVo, xhCRC) were seeded in ultra-low attachment 24-wells as described above. Recombinant human CEA (1000 ng/mL) were added every 3 days. After 6 days anchorage-independent culture, cell apoptosis was evaluated using FACS. CEA molecule protected CEA$^{-/lo}$ cells from anoikis, whereas exogenous CEA had little effects on CEA^{+} cells.

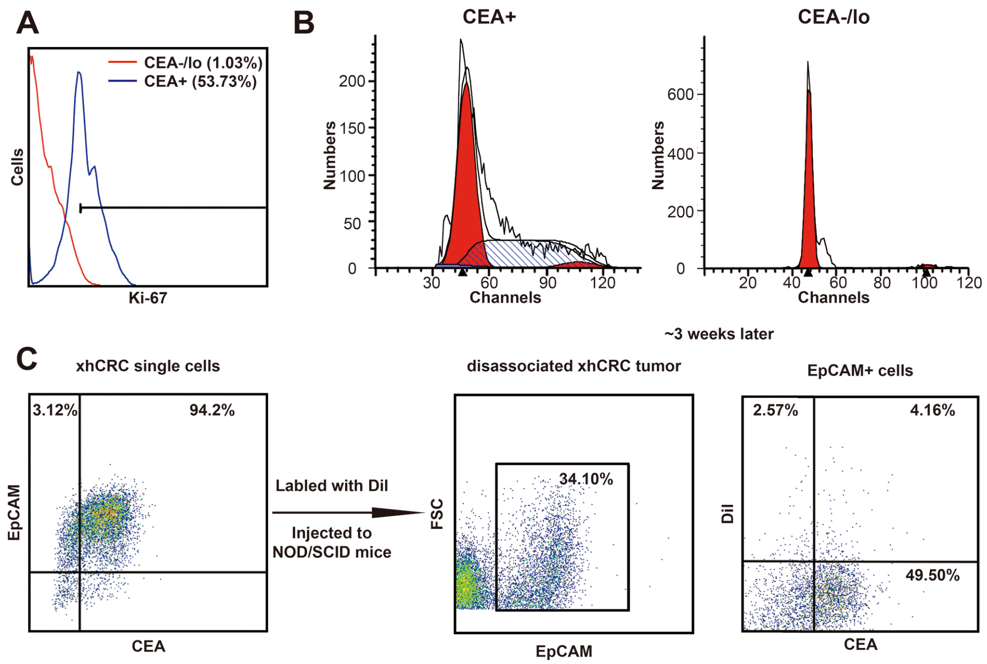

Supplementary Figure S5: CEA$^{-/lo}$ cells are relatively quiescent *in vivo*. (**A**) Ki-67 expression of purified CEA^{+} and CEA$^{-/lo}$ xhCRC cells. (**B**) Cell-cycle analysis of purified CEA^{+} and CEA$^{-/lo}$ xhCRC cells. (**C**) Flow cytometry analysis of DiI label retention in xhCRC after 3 weeks of chasing *in vivo*. Shown were the FACS profiles of CEA^{+} and CEA$^{-/lo}$ fractions in DiI^{+} cells. LRCs assays revealed that the percentage of CEA$^{-/lo}$ cells in Dil^{+} cells remarkable increased after implanted in NOD/SCID mice, suggested that CEA$^{-/lo}$ cells were quiescent *in vivo*.

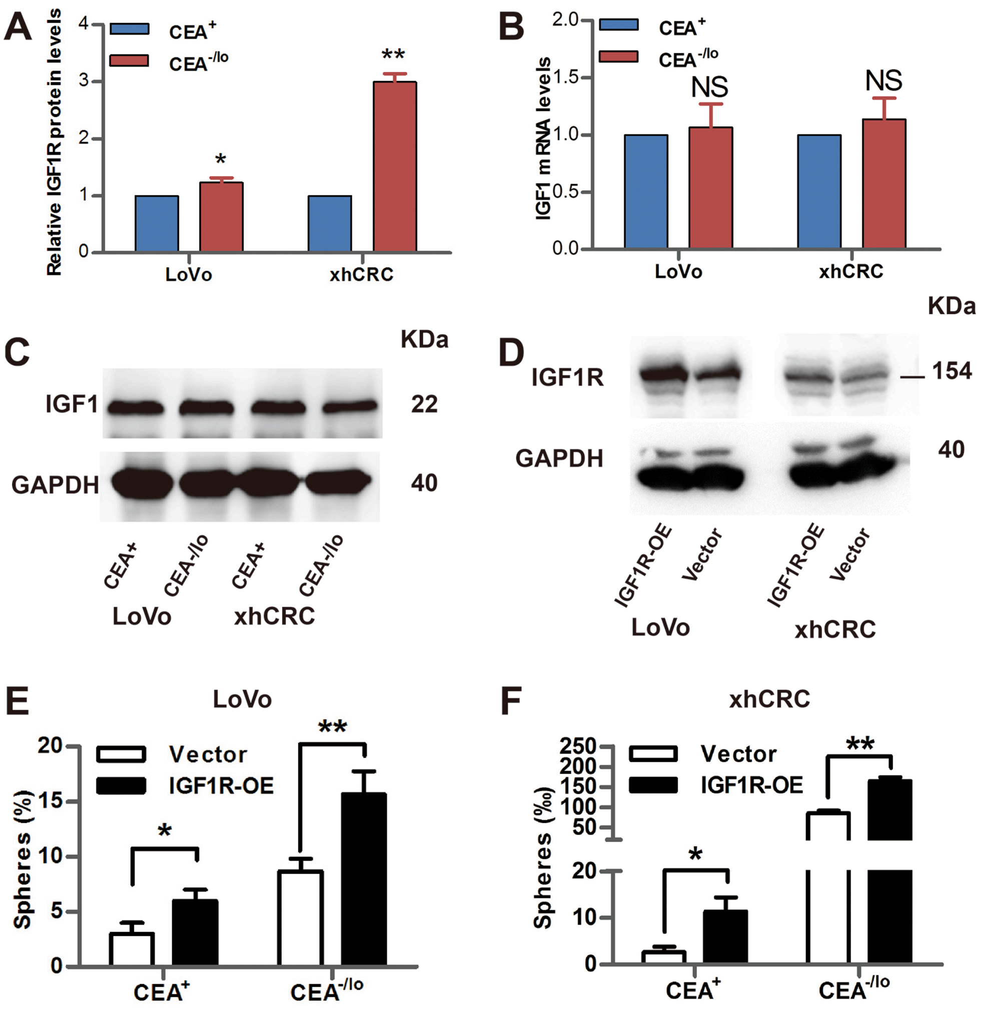

Supplementary Figure S6: IGF1R plays an important role in self-renewal of CRC cells. (**A**) Quantified western blot analysis on IGF1R expression in CEA^{+} and CEA$^{-/lo}$ CRC cells. Loading control was assessed by GAPDH. Data are represented as mean ± SD,*P < 0.05, **P < 0.01. (**B**) qPCR analysis of IGF1 mRNA levels in CEA^{+} and CEA$^{-/lo}$ cells. No significant difference (NS) was found. (**C**) Representative immunoblot analysis on IGF1 expression in CEA^{+} and CEA$^{-/lo}$ CRC cells. Loading control was assessed by GAPDH. (**D**) Immunoblot analysis of lentivirus-mediated IGF1R overexpression on CRC cells. Loading control was assessed by GAPDH. The 154KD IGF1R band was indicated with a short bar. (**E–F**) Sphere-formation assays of CEA^{+} and CEA$^{-/lo}$ cells infected by IGF1R-overexpressing lentivirus or vector. Data are represented as mean ± SD,*P < 0.05, **P < 0.01.

Supplementary Table S1: The histopathological classifications, serum CEA and proportions of $CEA^{-/lo}EpCAM^{+}$ cells of tumor tissue in patients with colorectal cancer

Case	Histology				Serum CEA (μg/L)	$CEA^{-/lo}$ (%)	Case	Histology				Serum CEA (μg/L)	$CEA^{-/lo}$ (%)
	Differentiation	T	N	M				Differentiation	T	N	M		
1	Well	3	0	0	0.97	53.44	22	Moderate	3	0	0	2.87	2.83
2	Moderate	4	0	0	3.46	21.93	23	Poor	4	x	0	20.96	93.02
3	Moderate	4	2	0	12.30	0.05	24	Moderate	4	0	0	4.9	16.03
4	Poor	3	0	0	4.96	6.13	25	Moderate	4	0	0	1.3	32.28
5	Moderate	4	1	0	4.72	0.00	26	Moderate	4	2	0	12.23	4.62
6	Poor	4	2	0	1.74	58.36	27	Poor	4	0	0	2.1	22.70
7	Moderate	2	2	0	21.21	55.90	28	Moderate	4	2	0	2.34	28.00
8	Moderate	4	2	0	2.01	60.18	29	Moderate	2	2	0	4.03	17.60
9	Moderate	2	0	0		51.60	30	Moderate	4	0	0	19.03	15.10
10	Moderate	2	0	0	1.67	38.34	31	Moderate	4	0	0	6.68	17.55
11	Moderate	4	0	0	4.79	44.65	32	Poor	4	2	0	21.39	70.00
12	Moderate	4	0	0	1.71	8.29	33	Poor	4	0	0	1.08	78.50
13	Well	2	0	0	2.83	33.04	34	Poor	4	0	0	13.81	36.28
14	Moderate	4	1	0	8.58	7.32	35	Poor	2	1	0	1.31	59.80
15	Moderate	2	0	0	5.99	19.72	36	Moderate	4	1	0	1.47	66.92
16	Well	1	0	0		0.66	37	Moderate	4	1	0	25.95	84.15
17	Well	4	0	0	1.67	9.41	38	Moderate	4	2	0	2.24	32.82
18	Moderate	4	2	0	1.23	5.46	39	Moderate	2	0	0	1.29	62.93
19	Moderate	4	1	0		24.46	40	Poor	4	0	0	8.69	27.48
20	Moderate	4	0	0	1.82	44.77	41	Moderate	4	0	0	2.37	31.82
21	Moderate	2	0	0	3.59	70.94							

Note. The proportions of $CEA^{-/lo}EpCAM^{+}$ cells in CRC tissues were evaluated using FACS. Tissues specimens were prepared into single cell suspension, and then cells were stained with propidium iodide, anti-CEA antibodies and anti-EpCAM antibodies.$PI^{-}CEA^{-/lo}EpCAM^{+}$ cells were defined as $CEA^{-/lo}$ cells.

Supplementary Table S2: Tumor incidence, latency and weights of CEA^{+} and $CEA^{-/lo}$ CRC cells in Balb/c-nu or NOD/SCID mice

Cell	Amount	Incidence	Latency (d)	Harvest (d)	Weight (mg)	*P* value
SW48	+10K	5/10	14	32	69.8 ± 52.6	< 0.05
	−10K	6/6	11	32	160.3 ± 75.9	
	+1K	4/10	14	32	49.5 ± 33.9	< 0.05
	−1K	6/8	11	32	141.7 ± 93.0	
LoVo	+10K	3/8	16	32	17.3 ± 1.7	< 0.05
	−10K	8/8*	13	32	91.3 ± 45.8	
	+1K	4/10	16	32	17.7 ± 2.5	< 0.05
	−1K	8/10	13	32	329.3 ± 229.5	
xhCRC	+1000K	6/10	17	31	85.2 ± 63.6	< 0.01
	−1000K	8/8	11	31	816.2 ± 199.7	
	+100K	6/10	22	31	59.7 ± 40.0	< 0.01
	−100K	9/10	16	31	501.7 ± 302.0	
	+10K	2/10	23	35	16.0 ± 8.5	< 0.05
	−10K	10/10*	17	35	71.6 ± 38.6	
	+1K	2/10	23	32	13.0 ± 0.0	< 0.05
	−1K	6/10	17	32	98.3 ± 103.2	
	+100	0/10		35		
	−100	3/10	22	35	104.7 ± 118.3	
	+10	0/10		35		
	−10	0/10		35		

CEA^{+} and $CEA^{-/lo}$ CRC cells were purified from SW48, LoVo and xhCRC, respectively. For each group, tumor incidence (*P <.05, Fisher exact test), time (days) of tumor latency and harvest, tumor weights (mean ± SD) and *P* values (Student *t*-test) were indicated.

Supplementary Table S3: Differential gene expression patterns in $CEA^{-/lo}$ vs. CEA^{+} xhCRC cells

	$CEA^{-/lo}$ cells	CEA^{+} cells
**(Cancer) stem cell & developmental molecules/markers**	CD44, BCR, IGF1R, IRS1, WNK1, FOXA3, WT1, NAB1, MED14, ITGB1, MXD1, KREMEN1, KIAA1217, HNMT, FGFR1OP2, EPPK1, ARL6	BASP1
**Differentiation-related**	DICER1, TMEM176B, MMD	MAF, QKI
**Metastasis/cell migration**	WNK1, MACC1, SYNE2, PTK2, PTPN12, E2F8, HS2ST1	
**Proteasome/ECM-related**	BAZ2B, SERAC1, NID1, RBPMS, C5orf55	
**Intermediate metabolism**	SULT1C4, C1orf43, UGCGL2, RDH13, GLS2, RETSAT, FH, OPA3, SLC35D1, PICALM, SYNJ1, GPX7, SLC38A6, CA14, OPLAH, UBE2Q1, RNLS, AK3, C8orf83, DAP2A, AGBL3, PDP2, ATP5S, MSMO1	ATP6V0D2, GOT2, ATP5O, HBE1, COX6A1
**Signal transduction**	SH3D20, ANKRD13C, QNL3L, CNIH3, GNL3L, GPR107, MAVS, PLEKHG4B, CCDC41, KCNE3,	AKT3, TBC1D8B
**Cytokines/inflammation and immune-related**	HEATR3, CTLA, TNFRSF11B, IL-11, IL-13RA1, IL10RB-AS1, ASB1, HLA-DMA,	SPG21, CD55, OAS2, PRLR, HLA-DR molecules, CIITA, TNFSF10, COL3A1, CXCL4, DDX58
**Transcription/nuclear factors**	ZNF713, ZNF207, ZNF341, ZNF268	ZNF479
**Cell adhesion**		CDH11, LGALS1
**Cell cycle**	CCNF, TUBCCP3, SDCCAG8, SKP2, NUDT6	CSPP1, RAN, PLK4, RBMS3, CCND2, POLA2
**Multi-drug resistance**	ABCC5, ABCG1, ABCB10	

Note. CEA^{+} and $CEA^{-/lo}$ cells were purified from xenograft tumor derived from CRC patient (xhCRC) and were applied for microarray analysis. Genes were considered differential expressed if the fold change was greater than 2.0 fold and the *P*-value was less than 0.05. Genes that play negative roles in regulation were marked in red.

Supplementary Table S4: Gene expression profiles both up-regulated in CEA$^{-/lo}$ LoVo and xhCRC cells

Symbol	Fold Change (CEA$^{-/lo}$ vs. CEA^{+})	
	LoVo	xhCRC
AK3	2.0133	2.0008
PANK2	2.4516	2.0040
MZT1	2.0194	2.0044
235286_at	2.0194	2.0072
MRPS12	2.6378	2.0383
TUG1	2.0877	2.0387
TSFM	2.1119	2.0644
BCR	2.2789	2.0648
IGF1R	2.0068	2.0907
NBPF3	2.2099	2.0947
FH	2.5387	2.1008
SELO	2.3452	2.1272
PAPL	2.0951	2.1580
WDR76	3.1397	2.1603
IL13RA1	2.0912	2.2311
TNRC6B	3.0285	2.2630
ASB1	2.2523	2.2989
LOC440288	2.6096	2.3207
RNASEH1	3.0798	2.3408
PTK2	2.3736	2.3866
RNF166	2.1331	2.4027
ALG10B	2.1336	2.4044
CCDC41	2.4414	2.4474
C1orf43	2.9647	2.4612
RDH13	3.1837	2.4969
SLC35D1	2.6638	2.6567
CYCS	2.9240	2.8161
CD44	2.5013	2.1915

Note. Genes were considered differential expressed if the fold change was greater than 2.0 fold and the *P*-value was less than 0.05. We compared differential expressed genes between LoVo cells and xhCRC cells.
